# Predictive Modeling Reveals Elevated Conductivity Relative to Background Levels in Freshwater Tributaries within the Chesapeake Bay Watershed, USA

**DOI:** 10.1021/acsestwater.4c00589

**Published:** 2024-10-30

**Authors:** Rosemary M. Fanelli, Joel Moore, Charles C. Stillwell, Andrew J. Sekellick, Richard H. Walker

**Affiliations:** † U.S. Geological Survey, South Atlantic Water Science Center, 3916 Sunset Ridge Road, Raleigh, North Carolina 27607, United States; ‡ Towson University, 8000 York Road, Towson, Maryland 21252, United States; § U.S. Geological Survey, MD-DE-DC Water Science Center, 5522 Research Park Drive, Catonsville, Maryland 21228, United States; ∥ University of Tennessee, 615 McCallie Ave, Chattanooga, Tennessee 37403, United States

**Keywords:** stream ecosystems, freshwater salinization, deicer applications, urbanization, water quality, random forests, Chesapeake Bay watershed

## Abstract

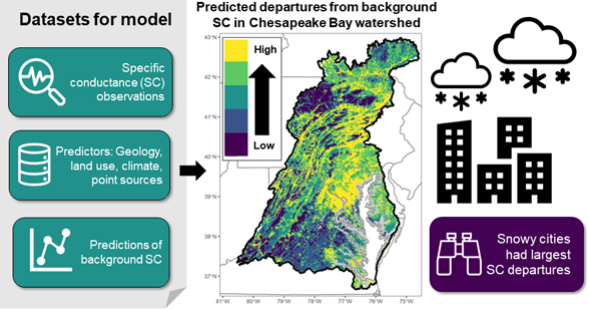

Elevated conductivity (i.e., specific conductance or SC) causes osmotic stress in freshwater aquatic organisms and may increase the toxicity of some contaminants. Indices of benthic macroinvertebrate integrity have declined in urban areas across the Chesapeake Bay watershed (CBW), and more information is needed about whether these declines may be due to elevated conductivity. A predictive SC model for the CBW was developed using monitoring data from the National Water Quality Portal. Predictor variables representing SC sources were compiled for nontidal reaches across the CBW. Random forests modeling was conducted to predict SC at four time periods (1999–2001, 2004–2006, 2009–2011, and 2014–2016), which were then compared to a national data set of background SC to quantify departures from background SC. Carbonate geology, impervious cover, forest cover, and snow depth were the most important variables for predicting SC. Observations and modeled results showed snow depth amplified the effect of impervious cover on SC. Elevated SC was predicted in two-thirds of reaches in the CBW, and these elevated conditions persisted over time in many areas. These results can be used in stressor identification assessments to prioritize future monitoring and to determine where management activities could be implemented to reduce salinization.

## Abstract 2

Increased conductivity is a stressor for freshwater organisms. This modeling study found that two-thirds of streams in the Chesapeake Bay watershed, USA, were predicted to have elevated conductivity originating from anthropogenic sources.

## 1 Introduction

Many freshwater ecosystems across the globe are experiencing increasing salinization.^1,2^ Elevated ion concentrations, often measured in aggregate as conductivity or specific conductance (SC, defined as conductivity at 25 °C), represent a substantial environmental stressor in freshwater ecosystems.^3−6^ Freshwater taxa are vulnerable to both direct and indirect effects of elevated conductivity and associated ions, with implications for the entire food web.^7−10^ For example, elevated chloride concentrations may enhance the toxicity on aquatic organisms of 6PPD, which is a chemical stabilizer found in automotive tires.^11^ Elevated conductivity and associated ions also alter biogeochemical cycling and increase mobilization of metals^12−17^ and may even disrupt natural sedimentation processes in freshwater streams.^18^ General declines in drinking water quality and an increased risk of corrosion within drinking water distribution systems have been documented in settings where dissolved ions are elevated.^19,20^

Conductivity in nontidal streams and rivers is influenced by natural and anthropogenic sources. Background conductivity (i.e., reference or natural levels not influenced by anthropogenic sources) is controlled by bedrock chemistry, precipitation inputs (quantity and chemistry), and evapotranspiration.^21,22^ Background conductivity differs among and within ecoregions given natural variability in these sources.^23^ Anthropogenic sources include point and nonpoint sources originating from urban and agricultural land uses, and especially winter deicer applications.^3,24,25^ Resource extraction activities (historical and current) also can elevate conductivity.^26^ Elevated SC can occur in reaches downstream of point source discharge locations^27^ or stormwater control measures.^28,29^ Urban development and agricultural intensification are among the primary drivers of reported increasing trends in conductivity and associated ions across the USA.^1,30−32^

Like many other temperate regions, the Chesapeake Bay watershed (CBW) has experienced extensive land use change, which may increase the salinization of freshwater ecosystems and impact freshwater biological communities. The CBW spans a five-state region within the USA (Pennsylvania, New York, Maryland, Delaware, and Virginia, including the District of Columbia) and is home to more than 18 million people with multiple growing metropolitan centers. A recent study predicted that about 30% of streams in the CBW have low Index of Biologic Integrity (IBI) scores for benthic macroinvertebrates.^33^ Low IBI scores were often located in urban settings, underscoring the need to assess potential in-stream stressors originating from urban development.^34^ Much of the CBW has naturally low background conductivity^22^ (<100 μS cm^–1^), but elevated conditions have been documented in areas with impervious surfaces and deicer applications.^35−37^ High chloride concentrations are a key cause of impairment in 28 watersheds across Maryland,^38^ and elevated salinity was identified as a key stressor driving biological impairment across the CBW.^6^ More information on the impacts of freshwater salinization in the region is needed to achieve ongoing restoration and conservation goals,^39^ especially since elevated conductivity may limit biological uplift following restoration.^40^

In this study, we applied a machine learning approach to (1) identify regional drivers of SC; (2) predict SC and departures from background SC in nontidal streams across the CBW; and (3) assess changes in these patterns over time. Agricultural land use and impervious surface cover were identified as primary anthropogenic drivers of elevated salinity in two recent national studies that examined freshwater salinization in stream ecosystems.^41,42^ National-scale studies are important for identifying general patterns in water quality but often lack the spatial resolution in both observations and predictor variables needed to identify subregional drivers. Moreover, neither study predicted SC conditions in unmonitored streams. Regional studies, by contrast, can leverage higher resolution data sets and be calibrated to regional conditions to provide more accurate results at management-relevant scales. This study used predictive modeling to estimate conditions in unmonitored reaches, which will fill a key information gap for managers in a region where coordinated efforts for ecological conservation and restoration are underway.^39^ Similar predictive studies have been completed for the CBW to characterize ecological flow alteration,^43^ physical habitat^44^ and biological conditions.^45,46^ The results from this study can help managers determine relative impairment from freshwater salinization within state-designated healthy watersheds, prioritize future monitoring needs, and determine if interventions could be implemented where SC is likely to contribute to biological impairment. These results may also provide additional context for field-based extirpation studies on salinity, which are often used to determine thresholds for biological impairment.^47^ More broadly, this study provides key insights into drivers of freshwater salination in temperate regions and provides a roadmap for predicting SC and departures from background SC in other regions.

## 2 Materials and Methods

### 2.1 General Approach

Median annual SC values and departures from background SC were predicted for nontidal stream reaches in the CBW. Publicly available SC data were compiled and spatially joined to a geospatial framework representing the CBW stream network. Watershed characteristics were compiled for the upstream accumulated area for each location with observations of median annual SC. A random forests model was developed and validated to predict median annual SC across the stream network. SC predictions were generated for four time periods using time-varying land use information: 1999–2001, 2004–2006, 2009–2011, and 2014–2016. Finally, a data set of modeled background SC was compared to predicted SC values to determine relative departures from background SC. Model input data, model output, and predicted SC values and departure class data sets are available online in a U.S. Geological Survey data release.^48^

### 2.2 Quantify Median Annual SC as Response Variable

All available SC observations located within the CBW were downloaded from the Water Quality Portal^49^ and screened, followed by unit harmonization to express SC in μS cm^–1^.^50^ Nontidal sites with a minimum of one SC observation per season for any year within each of the four time periods were included in the analysis (e.g., a site was eligible if it had one observation in all four seasons within the year 2001). Discrete SC observations were used to calculate median annual SC (μS cm^–1^) for the four time periods to represent the response variable for the model.

### 2.3 Geospatial Framework and Predictor Variables

The National Hydrography Data set Plus Version 2.1 (NHDPlusV2.1; 1:100K scale) provided the geospatial framework for this study.^51^ The NHDPlusV2.1 network for the CBW is composed of approximately 86,000 local catchments roughly 1–10 km^2^ in size, each with an associated flowline (i.e., stream reach). Stream reaches immediately adjacent to Chesapeake Bay were excluded, given the likely impact of tides or estuary salinity (see Supporting Information for more information). All selected sites with observational data (Section 2.2) were manually associated with the NHDPlusV2.1 network based on site coordinates. Watershed characteristics for the upslope accumulated areas for each site were compiled from sources already associated with the NHDPlusV2.1 network.^52,53^ An initial set of 45 predictor variables was selected and then pruned by examining pairwise correlations among possible predictor variables, using a correlation coefficient value of 0.7 as a threshold. This reduced the predictor variables list to 18 (plus time period) and included both static and time-varying variables describing land use, climate, geology, point sources, and physical watershed characteristics (Table SI-1 and SI text). Median annual SC values were paired with reach-specific predictor variables to form the records used in the random forest regression model (Table SI-2).

### 2.4 Random Forests Regression

The random forest (RF) algorithm is a machine learning method comprised of multiple uniquely generated decision trees.^54,55^ RF regression modeling and the assessment of predictor variable contributions were performed using R statistical software (version 4.1.3)^56^ with the following R packages: “ranger” (version 0.13.1),^57^ “tidymodels” (version 1.0.0),^58^ “DALEX” (version 2.4.3),^59^ and “DALEXtra” (version 2.2.1).^60^ An RF regression model was developed by using the compiled watershed characteristics as predictor variables (Table SI-1) and median annual SC values as the response variable (Table SI-2). Each record in the RF regression model represented a unique combination of site and time period (see SI for details). The model was trained and tested using a thrice-repeated 10-fold cross-validation and aggregation approach, resulting in 30 iterations from which model tuning and testing metrics were retained for evaluation. A final RF model was fit using all records yielding predicted median annual SC values (hereafter referred to as predicted SC) for nontidal CBW stream reaches for each of the four time periods. Exploratory analyses were conducted to estimate the relative importance of each predictor variable^54,61^ and feature contributions for each record.^62−64^ The relative importance of predictor variables was estimated by permuting predictor variable values across records, rerunning the model, and measuring the decrease in predictive accuracy.^54,61^ The contribution of each predictor variable value to a specific SC prediction was estimated using Shapley additive explanations (Shapley feature contributions, or SHAP values).^62,63^ The distribution of SHAP values was plotted for each predictor variable to understand the central tendency and contributions from each predictor variable to the modeled SC estimates.

### 2.5 Quantifying Departures from Background SC

Hydrological, water quality, and ecological studies often use measurements from undisturbed or least-disturbed stream reaches as benchmarks to which measurements in disturbed settings may be compared.^23,65,66^ We used one such method (quotient method) to assess deviations from “expected” conditions, for which predicted SC values from the RF model were divided by estimates of background SC values. Expected background SC values were calculated from an existing national data set comprised of predicted monthly background SC for the NHDPlusV2.1 network.^22^ This data set included monthly estimates of background SC for all NHDPlusV2.1 stream reach IDs in the USA from 2001 to 2015, which generally covered the study period. We calculated long-term average background SC using the monthly values from 2001 to 2015 to represent expected SC. This approach eliminated interannual variability from background SC in the quotient, which simplified comparison between departures from background SC and land use change over time.

Predicted/expected (P/E) ratios were generated by dividing the predicted SC values for each stream reach by its corresponding long-term background SC value. P/E ratios were then binned into five departure classes: (1) “At or below background SC” when the P/E ratio was ≤1; the predicted SC value was the same as or lower than background SC; (2) “1–1.5× background SC” when the P/E ratio was between 1 and 1.5; this category has greater uncertainty due to model error from this study and the model used to generate background SC estimates; (3) “1.5–2× background SC” when the P/E ratio was between 1.5 and 2; (4) “2–3× background SC” when the P/E ratio was between 2 and 3; and (5) “Greater than 3× background SC” when the P/E ratio was larger than 3. Predicted SC was generally considered elevated above background SC when P/E ratios were greater than 1.5.

### 2.6 Data Analysis

Predicted SC values, P/E ratios, and departure classes were summarized for the entire modeled area of the CBW and for areas comprising the nine major level III ecoregions in the CBW.^67^ Changes in SC departures were computed by subtracting the P/E ratio in the 2014–2016 time period from the 1999–2001 time period for all modeled stream reaches in the CBW. These changes in P/E ratios were binned as follows: (1) P/E ratio decreased by two or more; (2) P/E ratio decreased by 1–2; (3) P/E ratio decreased by less than 1; (4) little or no change in P/E ratio for stream reaches (i.e., less than a 10% change in the P/E ratio); (5) P/E ratio increased by less than 1; (6) P/E ratio increased by 1–2; and (7) P/E ratio increased by two or more. Finally, changes in P/E ratios were compared to changes in land use between the early and later time periods. All data processing and analysis were conducted using R version 4.2.1.

## 3 Results

### 3.1 Model Performance and Interpretation

**Figure 1:**
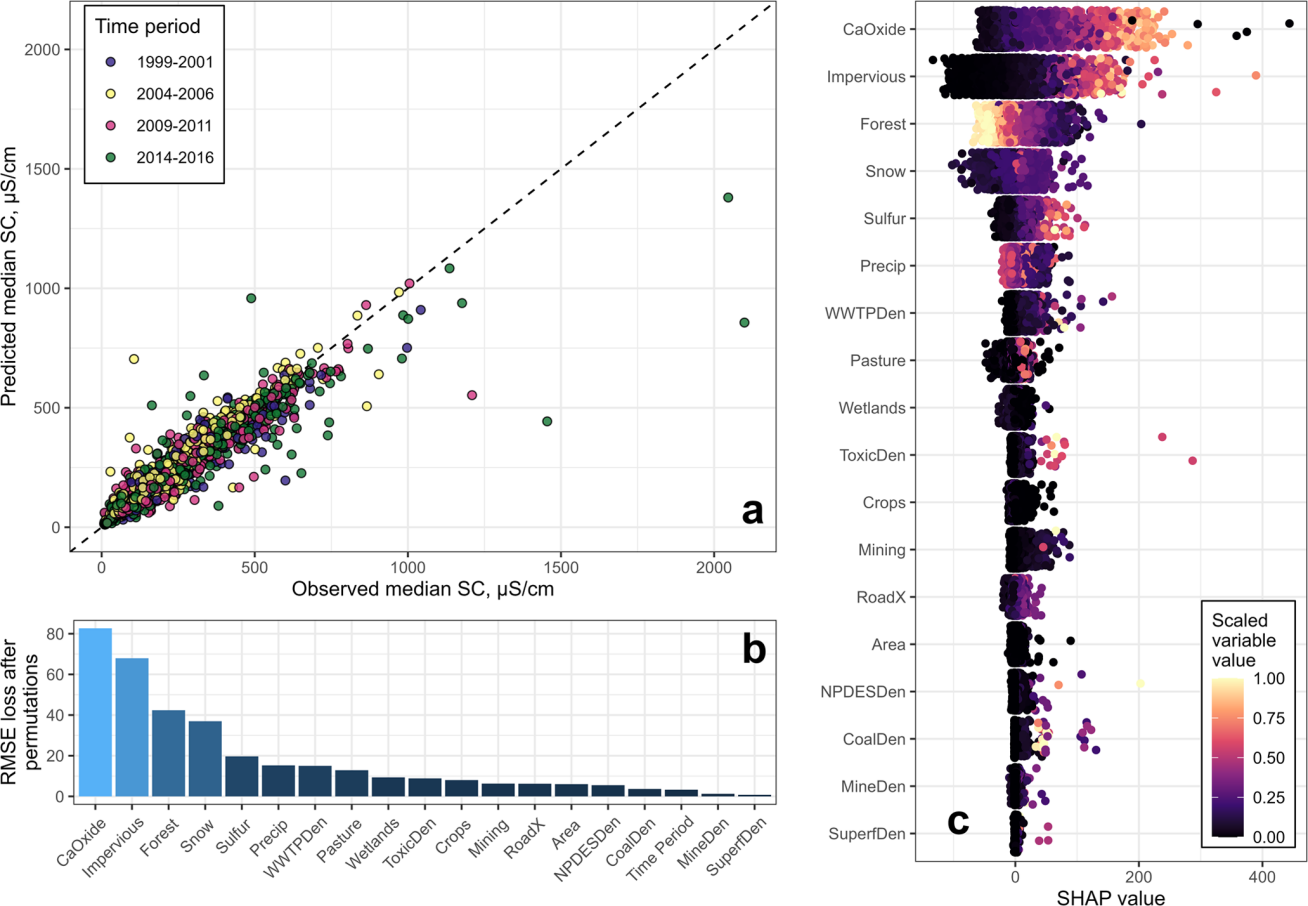
(a) Predicted versus observed median annual specific conductance (SC), μS cm^–1^, for the four time periods (*n* = 3,238 unique site/time period records). (b) Variable importance for the 19 predictor variables included in the random forests (RF) model. (c) Feature contributions (i.e., SHAP values) for each record in the RF model, colored by their scaled variable value (i.e., values converted to range between 0 and 1) and sorted by variable importance. For example, sites with low impervious cover (i.e., scaled variable values near 0) had negative SHAP values, meaning that predicted median annual SC for those sites was below the model-wide average predicted median annual SC. Forest cover had the opposite relationship (i.e., high forest cover had negative SHAP values). RMSE= root mean square error; CaOxide = percent mean lithological calcium oxide content; Impervious = percent impervious cover; Forest = percent forest land use; Snow = long-term average snow depth; Sulfur = percent mean lithological sulfur content; Precip = long-term average precipitation depth; WWTPden = wastewater treatment plant density; Pasture = pasture land use; Wetlands = percent wetland land use; ToxicDen = density of toxic release inventory sites; Crops = crop land use; Mining = percent mining land use; RoadX = density of road crossings; Area = watershed area; NPDESden = density of National Pollutant Discharge Elimination System sites; CoalDen = density of coal mine operations; Time period = three-year time window; MineDen = density of non-coal mine operations; SuperfDen = density of Superfund sites. See Table SI-1 for more details about variable definitions.

Locations with sufficient observations were spatially distributed throughout the CBW but with a greater density in Virginia and Maryland, especially in the later three time periods (Figure SI-1). There were 3,238 records and 1,708 sites (i.e., NHDPlusV2.1 stream reaches) included in the RF regression model. The number of records in each time period varied from 453 (1999–2001) to 1,017 (2009–2011; Table SI-2). Across the 30 testing iterations, the mean of mean absolute errors (MAE) was 38.4 μS cm^–1^ (95% confidence interval 36.9 to 39.8 μS cm^–1^), the mean of root-mean-square errors (RMSE) was 75.8 μS cm^–1^ (69.4 to 82.2 μS cm^–1^), and the mean of *r*-squared values was 0.811 (0.788–0.834). Median annual SC observations used in the model ranged from 8 to 2,099 μS cm^–1^, and predicted median annual SC varied from 11 to 1,380 μS cm^–1^ (Figure 1a). Ranges in predicted values were similar across the four time periods, although the mean, median, and maximum of SC records were highest in the last time period (Figure 1a; Table SI-2).

Two predictors describing natural SC sources were ranked as the first (percent lithologic calcium oxide content, or “CaOxide”; Table SI-1) and fifth (percent lithologic sulfur content, or “Sulfur”) most important variables for predicting SC across the CBW (Figure 1b). Percent impervious cover, percent forest cover, and long-term average snow depth were ranked as the second, third, and fourth most important predictor variables. Wastewater treatment plant density was the most important point source identified by the model (ranked seventh). The time period had the third lowest variable importance value, suggesting a lack of any unexplained variability across the four time periods.

Feature contributions (i.e., SHAP values) convey relative variable importance for individual predictions and illustrate both the magnitude and direction of the relationship between the predictor variables and median annual SC. In this model, SHAP values were positive and quite large (greater than 200 μS cm^–1^) for higher values of CaOxide, indicating a large, positive contribution to the SC prediction from CaOxide (Figures 1c and SI-2). By contrast, SHAP values were negative for high values of percent forest cover, indicating that forest cover reduced SC predictions. Impervious cover had a wide range of SHAP values with positive SHAP values occurring when impervious cover was greater than ∼ 2% (Figure SI-2).

**Figure 2:**
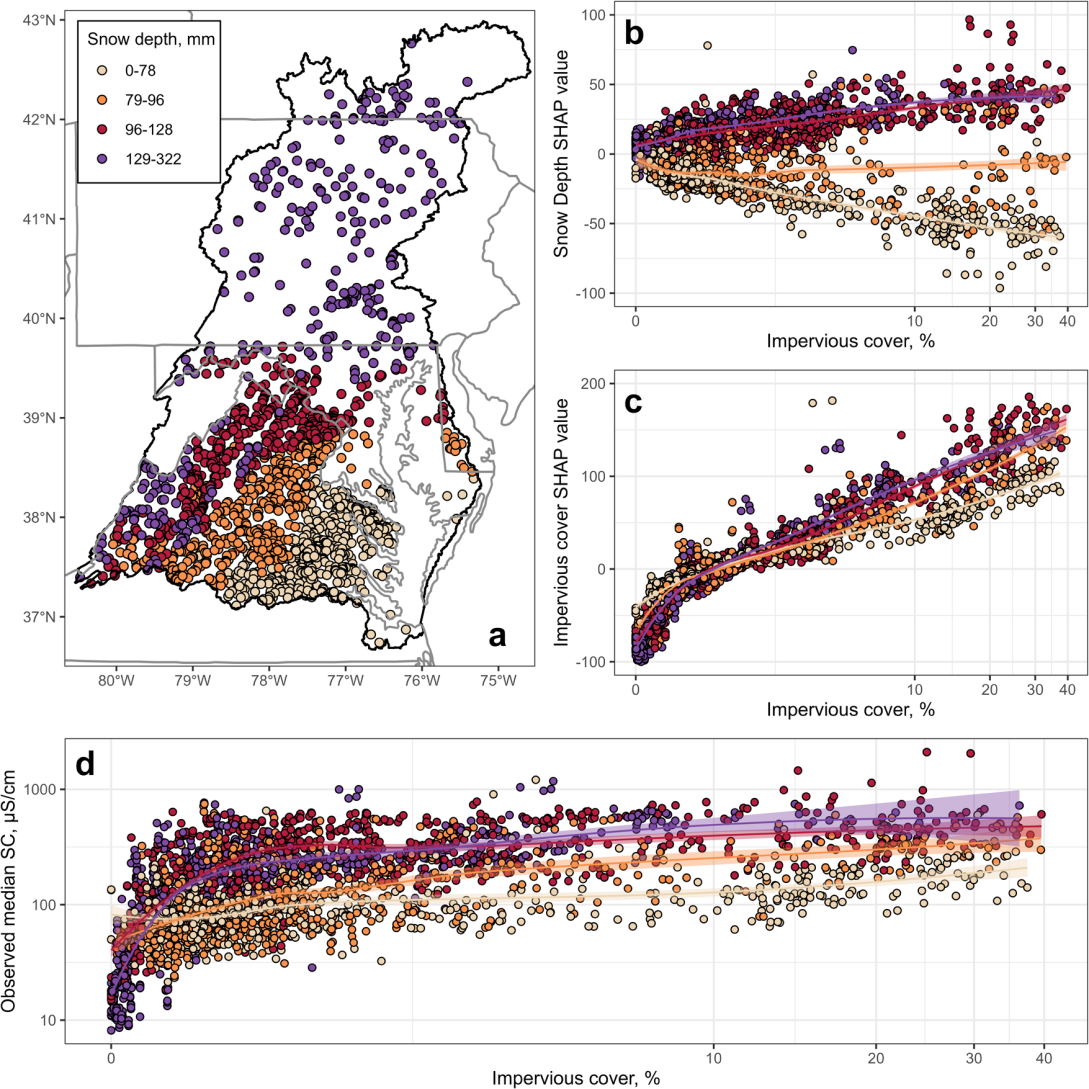
(a) Map showing spatial variability in the 2000–2014 average annual snow depth, mm, with snow depth grouped into quartiles (i.e., 0–25th percentile, 26–50th percentile, etc.) for all sites with specific conductance (SC) records used in the random forest model. (b) Snow depth SHAP values as a function of impervious cover and snow depth. (c) Impervious cover SHAP values as a function of impervious cover and snow depth. Note that the *y*-axis was limited to 200 μS cm^–1^ to better visualize the differences among snow quartiles, which excluded seven points from the figure. A full version of this figure can be found in Figure SI-3. (d) Observed median annual SC, μS cm^–1^, as a function of impervious cover and snow depth. Lines denote a loess smoothing function, and shaded areas around the lines represent 95% confidence intervals.

Reliable information about deicer material applications was not available for inclusion in the RF model, but the role of deicer applications on SC patterns was likely reflected by an interactive effect of snow depth with impervious cover. Snow depth increased with elevation and latitude across the CBW (Figure 2a). SHAP values for snow depth followed different patterns with increasing impervious cover depending on snow depth. For example, at sites with above-average snow depth (>96 mm), snow depth SHAP values were positive and increased with impervious cover, which contributed to higher overall SC predictions in these settings (Figure 2b). Snow depth SHAP values remained around zero or were negative with increasing impervious cover in areas with lower-than-average snow depth (<96 mm). Impervious cover SHAP values were higher at sites with greater snow depth (Figure 2c). This pattern was also apparent in the observed data (Figure 2d); for example, at 10% impervious cover, median annual SC was approximately 300 μS cm^–1^ higher for sites with more snow (129 mm or greater) than sites with less snow (less than 78 mm).

### 3.2 Basin-Scale Predictions of SC and Departures from Background SC

**Figure 3:**
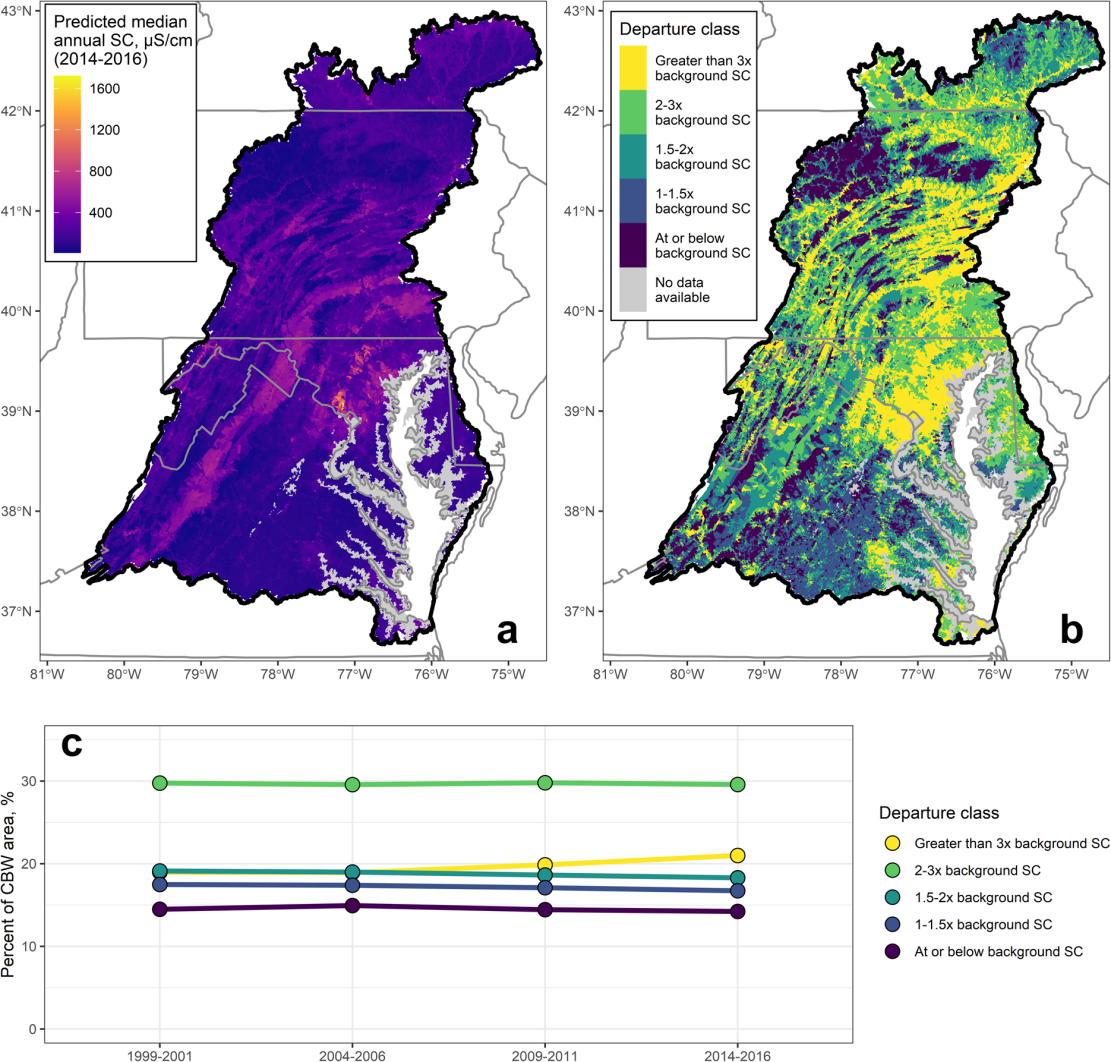
(a) Predicted median annual specific conductance (SC) for all modeled stream reaches in the Chesapeake Bay watershed (CBW) for the 2014–2016 time period. (b) SC departure classes for the 2014–2016 time period. (c) Percent of the modeled CBW area in each of the departure classes over the four time periods.

SC predictions were made for stream reaches whose local catchments represent 156,403 km^2^ of the CBW (92% of the land area). Areas with incomplete predictor data (35 km^2^) or close to Chesapeake Bay with presumed estuary influences (13,991 km^2^) were excluded from analysis. Long-term estimated background SC varied from 21 to 469 μS cm^–1^ (Figure SI-4a); higher background SC values occurred in areas with higher levels of CaOxide and Sulfur (Figure SI-5). Predicted SC across the CBW ranged from 10 to 1,720 μS cm^–1^ (Figures 3a and SI-4b). P/E ratios varied from near zero to 20.7, meaning that the predicted SC was over 20 times higher than the background SC at the highest end of the range (Figure SI-4c). The highest P/E values were concentrated around the Baltimore–Washington, DC corridor, and the lowest occurred in the northwest CBW in Pennsylvania and in central Virginia. Departure classes facilitate the visualization of P/E ratios (Figure 3b). Areas at or below background SC (i.e., P/E ratio less than or equal to 1) or slightly elevated/within model uncertainty (1–1.5× background SC) were mostly concentrated in central Virginia and in northwest CBW in Pennsylvania. Areas that were moderately elevated (2–3× background SC) or highly elevated (3× or more above background SC) were generally distributed throughout the upper two-thirds of the CBW.

Approximately 68% of the modeled area had predicted SC values greater than 1.5× background for the 2014–2016 time period, indicating widespread elevated conductivity throughout the CBW (Figure 3c; Table SI-3). The areas comprising the lower four departure classes decreased during the study period, including a 1.8% decline in the area with predicted SC levels at or below background SC. The total area with SC values 3× or more background SC increased by 9.6%. These patterns were likely driven by land-use changes across the basin during the study period (Table SI-4). At the beginning of the study period (1999–2001), the CBW was dominated by forest cover (61.2%), followed by agriculture (24%) and urban development (9.1%). Over the 15-year study period, forest cover declined 1.6% and agriculture declined 2.2%. Urban development increased by 6.6%. Mining land use increased by 15% (from 0.18% to 0.21% of the CBW) over the time period.

### 3.3 Patterns of SC Departure Classes across Ecoregions

**Figure 4:**
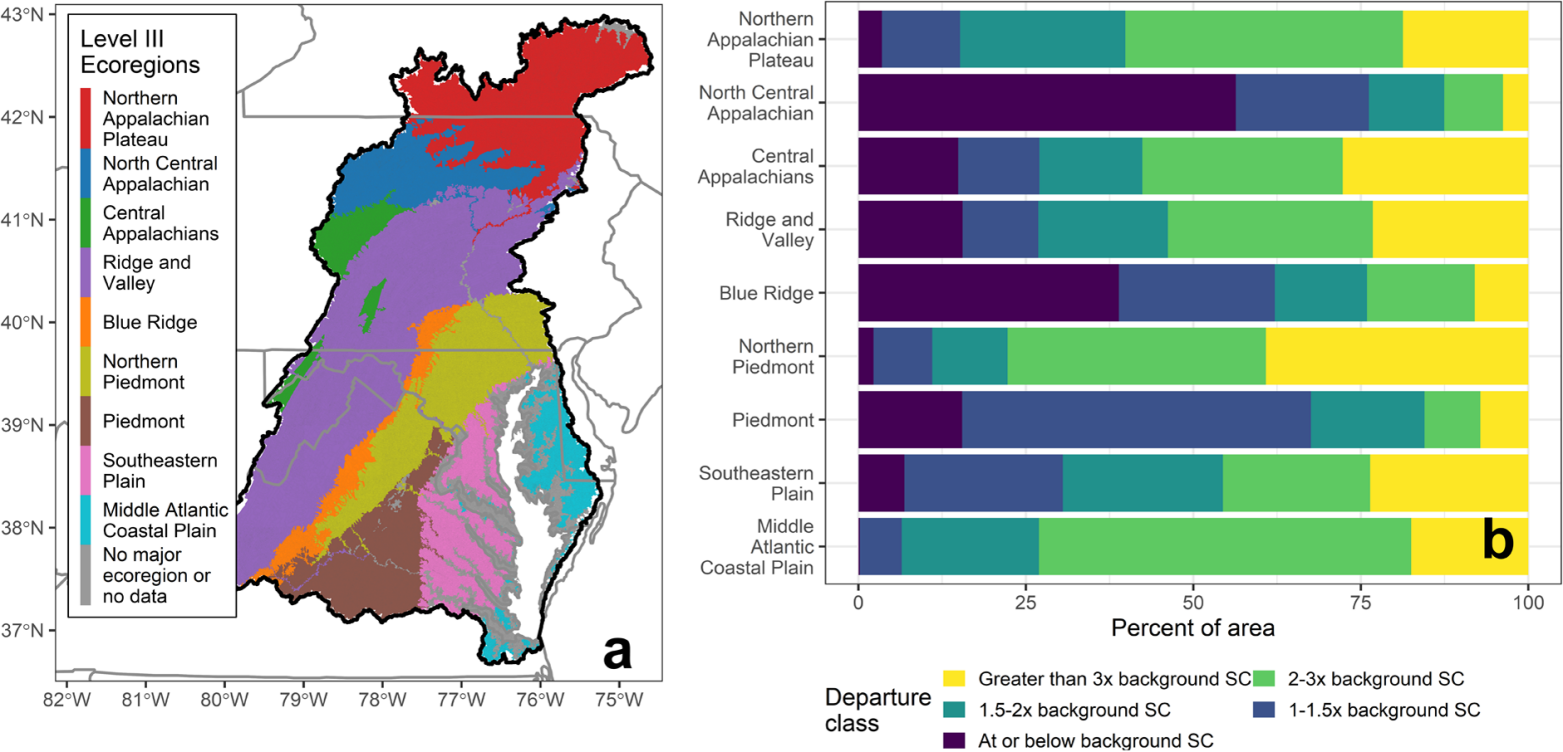
(a) Map showing the geographic extent of the nine major level III ecoregions. (b) Percent of the modeled Chesapeake Bay watershed (CBW) area in each of the specific conductance (SC) departure classes for the nine ecoregions during the 2014–2016 time period. Ecoregions are ordered from the north to the south.

Patterns in SC departure classes widely differed across the nine major level III ecoregions for 2014–2016 (Figure 4). The fewest SC departures occurred in the North Central Appalachian and Blue Ridge ecoregions, with 56% and 39% of reaches having predicted SC values at or below background, respectively (Figure 4b). These two ecoregions and the Piedmont had the smallest percent areas with highly elevated SC (3× or more the background SC; 3.8%, 8.0%, and 7.2%, respectively). In contrast, the Middle Atlantic Coastal Plain, Northern Piedmont, and Northern Appalachian Plateau had the smallest areas with predicted SC values at or below background SC (0.2%, 2.2%, and 3.5%). The Northern Piedmont and Central Appalachian ecoregions were heavily impacted by elevated SC, with SC values 3× background SC or more occurring in almost 40% and 28% of their respective areas.

These spatial patterns were likely driven by differences in land use across the nine ecoregions (Table SI-5). The North Central Appalachian and Blue Ridge ecoregions in the 2014–2016 time period had the largest share of forest cover (87% and 82%, respectively) and the smallest share of developed land use (3.2% and 5.2%, respectively). The Central Appalachians ecoregion had comparable levels of forest cover (77%) and urban land use (5.3%) to the Blue Ridge ecoregion, but 2.3% of its area was under mining land use in 2012 (an order of magnitude higher than the other ecoregions). The Southeastern Plain and Northern Piedmont ecoregions had the largest share of urban land use (19.9% and 19.7%, respectively). Row crops were most prevalent in the Middle Atlantic Coastal Plain (42%), followed by Northern Piedmont (18%). The Northern Piedmont and Northern Appalachian Plateau ecoregions also had extensive pasture land use (23% and 25%).

### 3.4 Changes in Predicted SC/Expected SC Ratios at the Ecoregion and Stream Reach Scales

**Figure 5:**
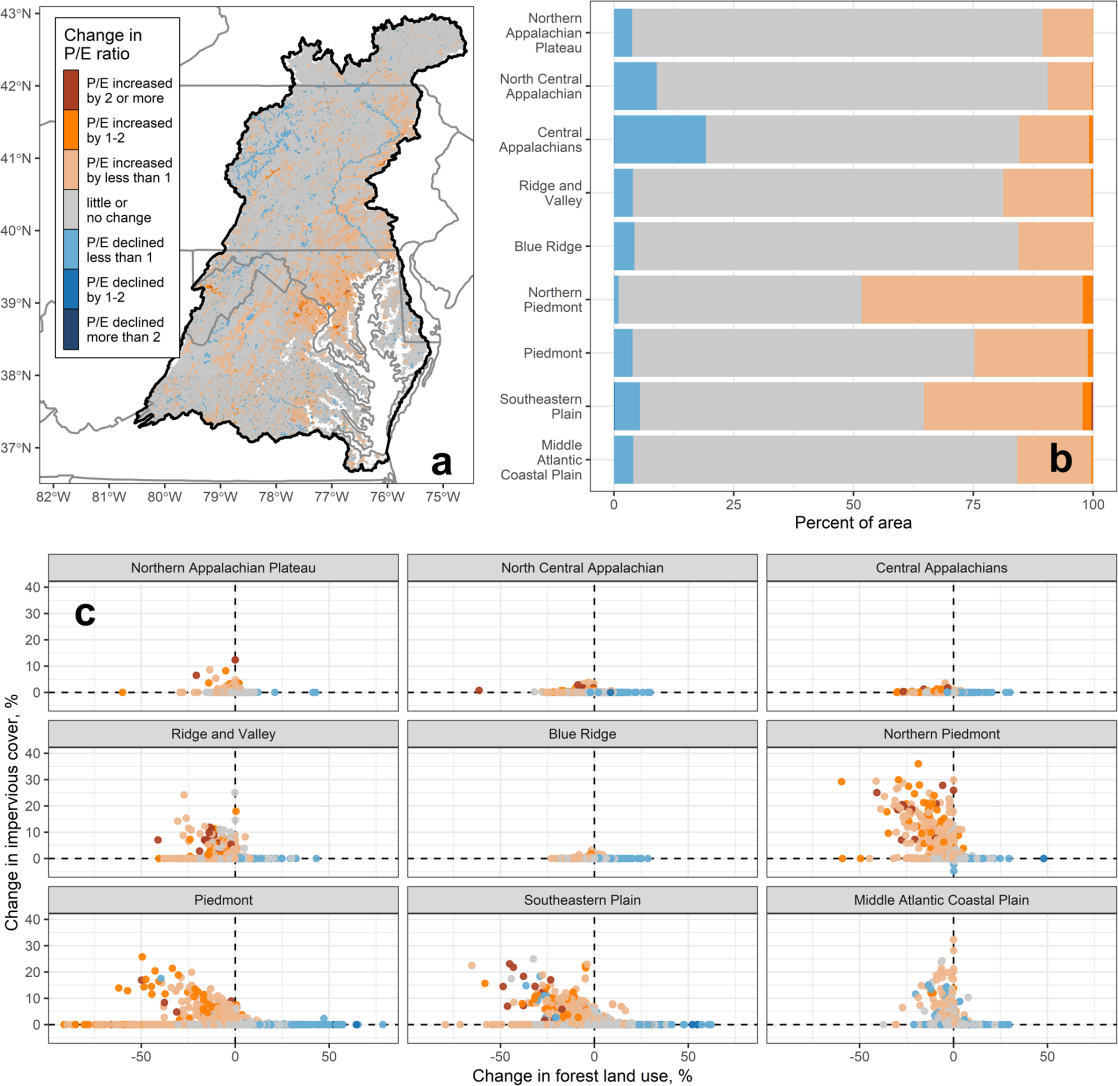
(a) Map of the change in the predicted specific conductance (SC)/expected SC (P/E) ratio from the 1999–2001 time period to the 2014–2016 time period for all modeled areas in the Chesapeake Bay Watershed (CBW). (b) Percent of the modeled CBW area in each of the P/E ratio change categories for the nine major ecoregions. (c) P/E ratio change categories for all stream reaches as a function of the change in percent impervious cover and percent forest land use during the study period.

Most of the CBW underwent little or no change in predicted SC/expected SC (P/E) ratios during the study period (Figure 5a), but some areas experienced modest changes in P/E ratios (an increase or decrease in the P/E ratio of less than 1). The Northern Piedmont and Southeastern Plain had the largest areas with increasing P/E ratios (48% and 35%, respectively; Figure 5b), and the largest magnitude increases in P/E ratios were concentrated in these two ecoregions as well. Few areas had declining P/E ratios in Northern Piedmont (less than 4%). The Central Appalachians ecoregion had the largest percent area where P/E ratios declined (∼20%), but this was mostly offset by another 15% of its area with increasing P/E ratios.

Land-use change varied widely among the nine ecoregions (Figure 5c). The largest increases in impervious cover occurred in Northern Piedmont; many of these stream reaches had increasing P/E ratios as well. Local catchments in the Piedmont and Southeastern Plain also underwent large increases in impervious cover (i.e., 10% or greater), where P/E ratios increased in many cases. In the Piedmont ecoregion, increased P/E ratios also occurred where forested cover showed large declines, but impervious cover remained constant. These large swings in forest land use without changes in impervious cover may be indicative of timber harvesting. Increases in forest cover exceeded 50% in some local Piedmont catchments, with declining P/E ratios for many of the associated stream reaches.

## 4 Discussion

Our study provided quantitative predictions of SC for four time periods to assess the spatial and temporal variability of SC and departures from background SC across the CBW. Two-thirds of freshwater stream reaches in the CBW were predicted to be impacted by elevated SC, and these conditions have persisted or worsened in many areas (Figures 3 and 5). Urbanization and deicer applications in snowier regions were the leading drivers of SC departures, but other anthropogenic sources also contributed to elevated SC conditions across the region. The SC departures data set provides a regionally relevant water quality benchmark, and this modeling approach can be applied in other regions to better understand the drivers of freshwater salinization. Region-specific data sets with high spatial resolution are essential for managers to assess relative impacts from freshwater salinization across different hydrogeological settings and ecoregions.^23^

A recent synthesis proposed five “state” factors that drive freshwater salinization: geology, climate, human activities, flow paths, and time.^68^ Our model results revealed that human activities (especially urbanization) and climate primarily controlled patterns of elevated SC in the CBW. These results somewhat differed from national-scale modeling studies on freshwater salinization.^41,42^ One study documented elevated SC in the mid-Atlantic and Northeastern USA, but the severity of the departures was less than what was revealed in this study, and agriculture was found to be a more important driver than urbanization.^41^ The other national study, which used sodium as a proxy for salinity, did identify urbanization as a major driver but did not differentiate between the effects of deicer applications vs other urban sources.^42^ A major advantage of regional-scale studies is the ability to better characterize and explore potential factors driving freshwater salinization that may only be revealed at smaller scales. For example, the inclusion of snow depth data to represent spatial climate gradients within the CBW region highlighted the varying effects of urbanization on SC due to differences in winter climate severity (Figure 2).

The highest median annual SC predictions occurred in urban areas receiving 96 mm of snowfall or more annually (Figure 2), contributing to the growing consensus that deicer applications on impervious surfaces are a major contributor to elevated salinity in regions with winter snow cover.^32,37,69−73^ Impervious cover and average snow depth were ranked as the second and fourth most important predictor variables in the RF model, respectively, and snow depth amplified the relationship between impervious cover and SC (Figure 2). Impervious cover is ubiquitous across the CBW (Figure SI-8), and increased SC occurred at low levels of impervious cover in this study (<5%; Figures SI-2 and SI-3). Similar levels of impervious cover have triggered increases in SC and chloride elsewhere.^24,74,75^ In northern regions, impervious surfaces receive variable amounts and types of deicer material depending on individual storm event characteristics, winter climate patterns, and municipal policies,^70,72,74^ which can lead to deicer loading to, and storage in, the subsurface.^28,76,77^ This process explains chronically elevated SC in the snowier ecoregions of the CBW (Figure 4). Over the study period, some northern ecoregions underwent substantial urban development (Figure 5), and future urban growth will likely exacerbate SC departures in those areas. Urbanization in southern ecoregions also increased SC likely due to deicer application from occasional winter weather events and other urban sources.

Other land uses influenced SC patterns. For example, intensive agricultural land use also likely contributed to elevated SC, as it had done so for chloride in lakes across the Northeast and Midwestern USA.^70^ Pasture and row crop land use were ranked 8th and 11th in the RF model, respectively (Figure 1b), and intensive agricultural land use was found in areas with high SC departures (Figure SI-8). Inorganic fertilizer and manure applications from animal operations are prevalent in these regions^78^ and are potential sources of excess ions. Groundwater monitoring found elevated SC in agricultural areas within the Middle Atlantic Coastal Plain of the CBW,^79^ suggesting long-term ion loading to the subsurface. Although SC levels for the Middle Atlantic Coastal Plain are elevated above the background (Figure 4b), they are not nearly as elevated as in urban centers, as seen throughout the Baltimore–Washington DC corridor (Figure 3b). Lower forest cover was also associated with elevated SC (Figure 1c and SI-2), likely due in part to replacement with other land uses. Some regions of the CBW show indications of active forest management during the study period (Figure 5), which may influence SC patterns. Logging activities, such as clearcutting or selective harvesting, commonly increase streamflow export and base cation leaching from soils,^80^ often leading to modest SC increases.^81,82^

Mining activities and point sources likely influence SC at local scales. Much of the CBW mining land use is in the Central Appalachians ecoregion (Table SI-5). Despite a large percentage of forest cover, the Central Appalachians have a low percentage of reaches at or below the background, and more than 25% of its reaches have highly elevated SC (Figure 4b), suggesting mining operations may increase SC in affected reaches. Coal mining and other energy extraction activities increase major ion and trace metal loading to receiving streams, which can substantially alter benthic macroinvertebrate community composition.^26,83^ Wastewater treatment plant density was ranked as most important among the point sources in the RF model and was, in general, evenly distributed across the ecoregions. These municipal and industrial sources can be sources of major ions and nutrients, both of which can contribute to elevated SC depending on the type and concentration of constituents being released.^27,84^

Natural sources (bedrock geochemistry) also strongly controlled SC patterns in the CBW. CaOxide and Sulfur were ranked as the first and fifth most important predictor variables in our RF model, respectively (Figure 1) and were also the top two predictors of background SC across the USA.^22^ Monitoring of streams, wells, and springs located in carbonate settings with little to no anthropogenic inputs revealed high SC values within the CBW (Table SI-6 and Figure SI-9). Streams underlain by carbonate bedrock have naturally high SC^85,86^ because carbonate mineral dissolution often dominates stream chemistry even when comprising only 1–3% of bedrock in some settings.^87,88^ These naturally elevated SC levels can make it difficult to accurately assess departures from reference conditions across large, diverse regions.^23^ Yet, characterizing the extent and severity of water quality levels above ecological benchmarks is necessary for causal assessments to identify sources of biological impairment.^7^

The only current EPA-established SC benchmark for freshwater aquatic life is 300 μS cm^–1^ for the Central Appalachian ecoregion.^89^ The national background SC data set^22^ used in this study is an improvement over this benchmark because it provides a flexible baseline to account for natural variability in background SC, which is important given that tolerance to salinity and major ions varies across taxa and prevailing background conditions.^90^ However, analysis of the input data for the background SC model indicated that carbonate settings were likely underrepresented in their analysis (Figure SI-10). This underrepresentation could lead to underprediction of background estimates for these areas (Figure SI-9a), thereby erroneously flagging these reaches as elevated. Further analysis of empirical SC data confirmed background SC in carbonate settings is likely higher than predicted in the background data set (Figure SI-9b). Experimental evidence points to SC increases having more deleterious effects on organisms from naturally low SC streams, particularly macroinvertebrates, than on organisms from naturally high SC streams.^90,91^ The quotient method we applied to determine departures is more sensitive at low background SC and more forgiving at high background SC (Figure SI-11), which addresses this variability in the sensitivity of taxa to changes in SC. However, there could be some cases in which unimpaired carbonate streams have been flagged as elevated in our analysis. Managers working in carbonate settings may consider conducting local assessments of background SC.

Data availability influenced our study design and the results. First, sites that fit our study criteria were not evenly distributed across the CBW (Figure SI-1), so some settings were underrepresented in the model. For example, SC observations were not available in Piedmont areas that underwent large changes in forest land use, so we were unable to verify the potential effect of timber harvesting on SC. Second, our response variable was based on a minimum of four observations per year and, therefore, cannot be expected to predict transient in-stream conditions (i.e., short-term SC increases following deicer applications). However, median annual chloride values were found to be correlated with aquatic life exceedance durations for chloride in the region,^37^ suggesting annual-scale metrics may be sufficient for detecting general levels of impairment.

Our model included a limited number of time-varying predictor variables (Table SI-1). Of the top five variables in the RF model, two were time-varying (percent forest and percent impervious) and two were static geological variables (CaOxide and Sulfur). The fifth variable, snow depth, for which we used a long-term average, reflected the average relative severity of winter seasons across the CBW. Since snow depth (and associated deicer application) fluctuates among years, including a time-varying winter snow depth or winter severity variable may improve model prediction resolution during individual periods. Unfortunately, data describing interannual snow depth were not available at the time of the study. Although we did not have data to characterize snow depth for these periods across the basin, we intentionally used three-year windows to represent each time period to minimize the effects of a single high or low snow year. Both snow depth and impervious cover are associated with deicer applications, but impervious cover is associated with many additional sources of SC as well. Since impervious cover has almost twice the variable importance of snow depth (Figure 1), we believe it has a larger effect on SC patterns in the region than snow depth. The mine density and point source variables included in the RF model were also static, which likely does not reflect variable discharge volumes over time. Finally, we did not consider other minor inputs, such as coastal aerosols, which may have a small influence on nontidal stream reaches close to the Bay.

## 5 Conclusions

Freshwater salinization has become a considerable threat to stream ecosystems and water supplies globally. Our study aimed to identify key drivers of freshwater salinization and predict median annual SC conditions in unmonitored streams throughout the temperate CBW region. This study is the first regional-scale predictive modeling assessment of stream SC and reveals that key drivers and the extent of elevated SC within the CBW differ from a previous national study.^41^ Elevated SC and departures from background SC were widespread across much of the CBW. Impervious cover in association with greater snow depth was the largest factor driving elevated SC in the CBW. Other factors such as agriculture, decreased forest cover, or mining also played important roles within some ecoregions as well (e.g., deforestation and reforestation in the Piedmont, mining in the Central Appalachians). These findings are relevant for other temperate regions, such as the Northeast and Midwestern USA and Canada, where similar settings exist. Predictions from our model can be directly used to identify potential biological stressors, highlight monitoring gaps, and manage sources of salinity. This modeling approach can also be applied elsewhere to assess the relative importance of different factors driving freshwater salinization.^68^ Finally, this study can serve as a blueprint for assessing thresholds in water quality conditions in other regions where stream ecosystems are at risk of impairment.

## Data Availability

Model input data, model output, and predicted SC values and departure class data sets are available online in a U.S. Geological Survey data release.^48^

## Supporting Information Available

Additional methods details regarding quantifying median annual SC as response variable; geospatial framework and predictor variables; random forests regression; quantifying departures from background SC; and data analysis (PDF)
Predictor variables considered or used in the random forest model (XLSX)
