# Supplementary material for: Predictive Modeling Reveals Elevated Conductivity Relative to Background Levels in Freshwater Tributaries within the Chesapeake Bay Watershed, USA
Source: ACS ES T Water. 2024 Oct 30;4(11):4978–89. doi: 10.1021/acsestwater.4c00589 (PMC11555677; doi:10.1021/acsestwater.4c00589)
Supplement: Supplementary file 1 — ew4c00589_si_001.pdf [file ew4c00589_si_001.pdf]

Supplemental information for:

"Predictive modeling reveals elevated conductivity relative to background levels in freshwater tributaries within the Chesapeake Bay watershed, USA"

Authors: Rosemary M. Fanelli[1*], Joel Moore[2], Charles C. Stillwell[1], Andrew J. Sekellick[3], and Richard H. Walker[4]

[1]U.S. Geological Survey South Atlantic Water Science Center, Raleigh, NC

[2]Towson University, Towson, MD

[3]U.S. Geological Survey MD-DE-DC Water Science Center, Catonsville, MD

[4]University of Tennessee, Chattanooga, TN

*Corresponding author (rfanelli@usgs.gov)

Additional methods details

Quantify median annual specific conductance (SC) as response variable. Sites with presumed tidal influences were excluded from the analysis. Tidal influence was defined as being located within identified NOAA Chesapeake Bay segments (Chesapeake Bay Program, 2004). Seasons for determining site eligibility were defined as follows: Winter = Dec, Jan, Feb; Spring = Mar, Apr, May; Summer = Jun, Jul, Aug; Fall = Sept, Oct, Nov. Sites with SC observations were initially assigned to the closest NHDPlusV2.1 flowline reach ID (stream reach). Next, all NHDPlusV2.1 assignments were manually checked and visually verified to ensure accuracy and corrections were made if needed. The last observation screen step was to filter duplicate sites that were located on the same NHDPlusV2.1 stream reach. For sites on the same stream reach with similar medians (<50 µS/cm difference), one site was randomly selected to be included in the random forests model. Highly variable observations located along the same reach (medians that differed by ≥50 µS/cm) were dropped from the analysis.

Geospatial framework and predictor variables. The National Hydrography Dataset Plus Version 2.1 (NHD) served as the geospatial framework for this study. Analysis excluded any NHD stream reaches with presumed tidal influences, which were identified by their location within identified NOAA Chesapeake Bay segments (Chesapeake Bay Program, 2004).

We calculated pairwise correlation coefficients (CC) to trim the initial list of 45 variables and used 0.7 as a threshold to indicate high correlation. Variables were grouped by topic and their CCs were examined simultaneously (for example, variables describing urbanization included percent impervious cover in the watershed, percent impervious cover in the riparian zone, percent developed land use, road density and road crossings). Many variables within a topic were highly correlated, so only 1-2 were selected to represent each group. We used *a priori* knowledge of water quality, hydrology, and freshwater salinization to facilitate variable selection within a group. For example, we initially considered three climate variables for the model: 1) long-term average annual snow depth, 2) long-term average annual precipitation, and 3) long-term average annual air temperature. Annual snow depth and annual air temperature were highly correlated (CC > 0.7). Snow depth was selected because its potential importance was

supported by existing research linking snowfall with deicer applications. Neither snowfall nor temperature were highly correlated with long-term average precipitation, however, so precipitation remained in the final model.

Land use variables reflected dynamic conditions in the final random forest model. Percent land use classes (or percent impervious cover) varied across the four time periods to represent major National Land Cover Database (NLCD) years (2001, 2006, 2011, and 2016; Dewitz, 2021). Percent mining also varied over time. The 2002 National Water-Quality Assessment's Wall-to-Wall Anthropogenic Land Use Trends (NWALT) dataset was used to reflect conditions in the first and second time periods, and the 2012 NWALT dataset was used to reflect conditions in the latter two time periods (Falcone, 2015). Static variables included additional indicators of urbanization (road density, road crossings), indicators of long-term climate (average annual precipitation and snow depths), geological sources of salinity (percent lithologic calcium and sulfur content of bedrock).

Random Forests Regression. We defined a *single record* in the random forest (RF) model as each unique combination of site (stream reach) and time period (one of 1999-2001, 2004-2006, 2009-2011, and 2014-2016). Each record's SC value represented the median of all available SC observations from a given year in the time period. If a site had multiple years with eligible SC data in a time period (*e.g.* 1999 and 2000), one year's median SC was randomly selected for the record. Table SI-2 contains summary information on the SC records used in the final RF model.

Each site was considered independent from other sites. If SC data from multiple time periods were available at a single site, then those records were not considered independent because the static predictor variables were identical and most of the time-varying predictors, while not identical, were likely to be similar across time periods. Replicate records, or in this case, near-replicate records, affect the random forest algorithm (and underlying regression trees) by implicitly adding weight to the duplicated values (Toth and Eltinge, 2011; Nalenz and others, 2024). Although each individual record was equally likely to be randomly selected during each regression tree's random sampling from the training dataset, a site with SC data from all four time periods was four times as likely to be represented in the training set than a site with SC data from only a single time period. To ensure that all sites were equally likely to be represented in training, records were weighted based on the inverse of the number of time periods available per site. For instance, if a site had SC data during a single time period, it was assigned a weight of 1.0; conversely, if a site had SC data during all four time periods, each record was assigned a weight of 0.25. The assignment of record weights prevented the RF model from overfitting to the sites with multiple records. Furthermore, records from the same site were grouped together to prevent the possibility of near-replicate records from being split into both the training and testing sets (and subsequently inflating accuracy metrics derived from the test set; Toth and Eltinge, 2011; Nalenz and others, 2024). Stratified sampling was utilized to balance the representation of carbonate sites (calcium oxide content, or CaOxide, ≥15%) and non-carbonate across training and testing sets.

Model hyperparameters 'mtry' (number of predictor variables to consider at each node), 'num.trees' (number of regression trees per RF), and 'min.node.size' (minimum node size) were tuned during each training iteration. The RF hyperparameter tuning procedure resulted in a 'mtry' of 9, 'num.trees' of 1,000, and 'min.node.size' of 5.

Quantifying departures from background SC. Expected natural background SC values were derived from a study that spanned the continental United States (Olson and Cormier 2019). They used the same geospatial framework, but selected sites with little or no anthropogenic influence on model factors that control background SC. The Chesapeake Bay watershed (CBW) portion of the Olson and Cormier (2019) input dataset used to develop their model did not include observations for reaches where CaOxide was ≥25% and few with CaOxide ≥15% (Figure SI-10). Reference sites included in the Olson and Cormier (2019) input dataset were required to have little to no agricultural land cover, which precluded inclusion of high carbonate sites in the CBW. Agricultural land cover, particularly pasture, is widespread in regions of the CBW with carbonate bedrock, particularly the Ridge and Valley ecoregion (Figures 4 and SI-8) that extends across Pennsylvania, Maryland, Virginia, and West Virginia. Regions underlain by carbonate are in valleys and have likely been used for agriculture for more than a century. Thus, carbonate reaches in the CBW were not available to serve as reference sites in Olson and Cormier (2019).

To improve understanding of background SC in carbonate settings, we compiled SC data for carbonate sites (CaOxide ≥15%) from other sources, most of which are within the CBW (Bolton, 1998; Calmels et al., 2014; Hyer et al., 2016; Krawczyk and Ford, 2006; Nelms and Moberg, 2010; Vesper and Herman, 2020). Geochemical data for CBW stream and spring sites with ~10–40% CaOxide, major ion concentrations, and differing degrees of anthropogenic influence were included in the compilation. Determining precise background values is challenging because even in a “pristine” system, SC across carbonate sites in the CBW can differ by as much as 200 µS cm^{-1} depending on the bedrock chemistry (the mix of limestone and dolostone) and on the hydrologic routing of the karst system (Langmuir, 1971). Anthropogenic influences on stream geochemistry will add further variability. Expected background SC values for CBW specific reaches as modeled by Olson and Cormier (2019) were obtained via the US EPA Freshwater Explorer (Cormier et al., 2021) for comparison to observed SC values for carbonate CBW sites.

SC values for 18 carbonate stream and spring sites from 5 CBW locations were 90–460 µS cm^{-1} higher than the "expected” Olson and Cormier (2019) background values for sites (Figure SI-9, Table SI-6). The maximum predicted background SC values from Olson and Cormier (2019) for any of these reaches was 323 µS cm^{-1} and many “expected” background values are much lower (Table SI-6). Forested carbonate watersheds in France (CaOxide ≈ 40% based on calcium carbonate stoichiometry) have similar SC values to minimally perturbed carbonate sites in the CBW (Figure SI-9B). Large differences between actual and “expected” SC occurred even at several sites where <10% of the anion charge (µeq L^{-1}) originated from chloride, nitrate, and sulfate (Figure SI-9A, Table SI-6). The 10% threshold has been suggested to represent minimal anthropogenic influence in carbonate springs and streams (Krawczyk and Ford, 2006). “Expected” background SC values show little change as bedrock CaOxide increased from 10 to 20% and then expected SC slowly increased in a generally linear pattern from ~20–35% CaOxide (Figure SI-9B). It is more likely that increases in SC due to higher subsurface calcium occur as a step function since carbonate minerals dissolve several orders of magnitude faster than silicate minerals (Lerman and Wu, 2008).

Several simplifying assumptions were made when interpreting the compiled carbonate stream and spring data. First, spring and stream results can be combined. Second, the CaOxide values from Olson and Cormier (2019) for stream reaches are the same or similar for adjacent

springs. Third, the CaOxide value for a stream reach is the same for all sites along that reach. In two cases, multiple sites in Virginia were included from the same NHDPlusV2.1 stream reach: (1) two sites (stream and adjacent spring) from a Spring Creek, VA stream reach with a CaOxide value of 34.4% and (2) four sites (three stream and an adjacent spring) from War Branch, a tributary of Smith Creek, VA with a CaOxide value of 24.5%. Fourth, for the French data, which are from forested watersheds with minimal anthropogenic influence (Calmels et al., 2014), the percent of CaOxide was estimated assuming that the bedrock was pure limestone (40% Ca, plotted at 39.5 CaOxide). SC values for Calmels et al. (2014) were calculated using ion concentrations and PHREEQ, which is a geochemical mixing model (Parkhurst and Appelo, 2013; McCleskey, 2018) with results for sites <650 m elevation included herein. Sites <650 m were selected because of the similarity to elevations of the CBW carbonate sites, and Calmels et al. (2014) found that SC decreased with elevation, likely due to decreased soil CO_2 driven by vegetation.

SC values can change over time due to increased anthropogenic influence. For example, Muddy Creek, Virginia (CaOxide = 29.56%, Fig. SI-9b) has USGS major ion data from 1993 to 2022. Between the periods 1993–97 and 2018–22, median SC increased from 424 to 504 µS cm^{-1} and the median charge contributed by chloride, nitrate, and sulfate increased from 14.8 to 18.1% of anion charge and from 748 to 1131 µeq L^{-1} (Table SI-6). SC values in carbonate springs with substantial inputs from agricultural or urban landscapes range from 700 to >1500 µS cm^{-1} (Toran et al., 2006, unreported data from springs studied in Toran et al., 2009 (Dr. Laura Toran, Temple University, written commun., March 4, 2022)).

Data analysis, additional detail. Departures from background SC were summarized for major level III ecoregions for the study. Although there are 12 level III ecoregions present within the CBW, three cover only a small fraction of the basin; the nine major ecoregions comprise more than 99% of the study area. As such, the three remaining ecoregions were dropped from that portion of the analysis. The three minor ecoregions dropped were Eastern Great Lakes and Hudson Lowlands, Northeastern Highlands, and Western Allegheny Plateau, which respectively comprise 585 km^2, 13km^2 and 4 km^2 of the study area (0.37%, 0.01% and 0.01%). A dominant ecoregion was assigned to each reach by calculating the percentage of each ecoregion present in the upstream accumulated area (i.e., their drainage area). An ecoregion was assigned if one comprised 50% or more of its upslope accumulated area. Only 648 km^2 of the study area (0.41%) had no dominant ecoregion; these reaches were dropped from that portion of the analysis.

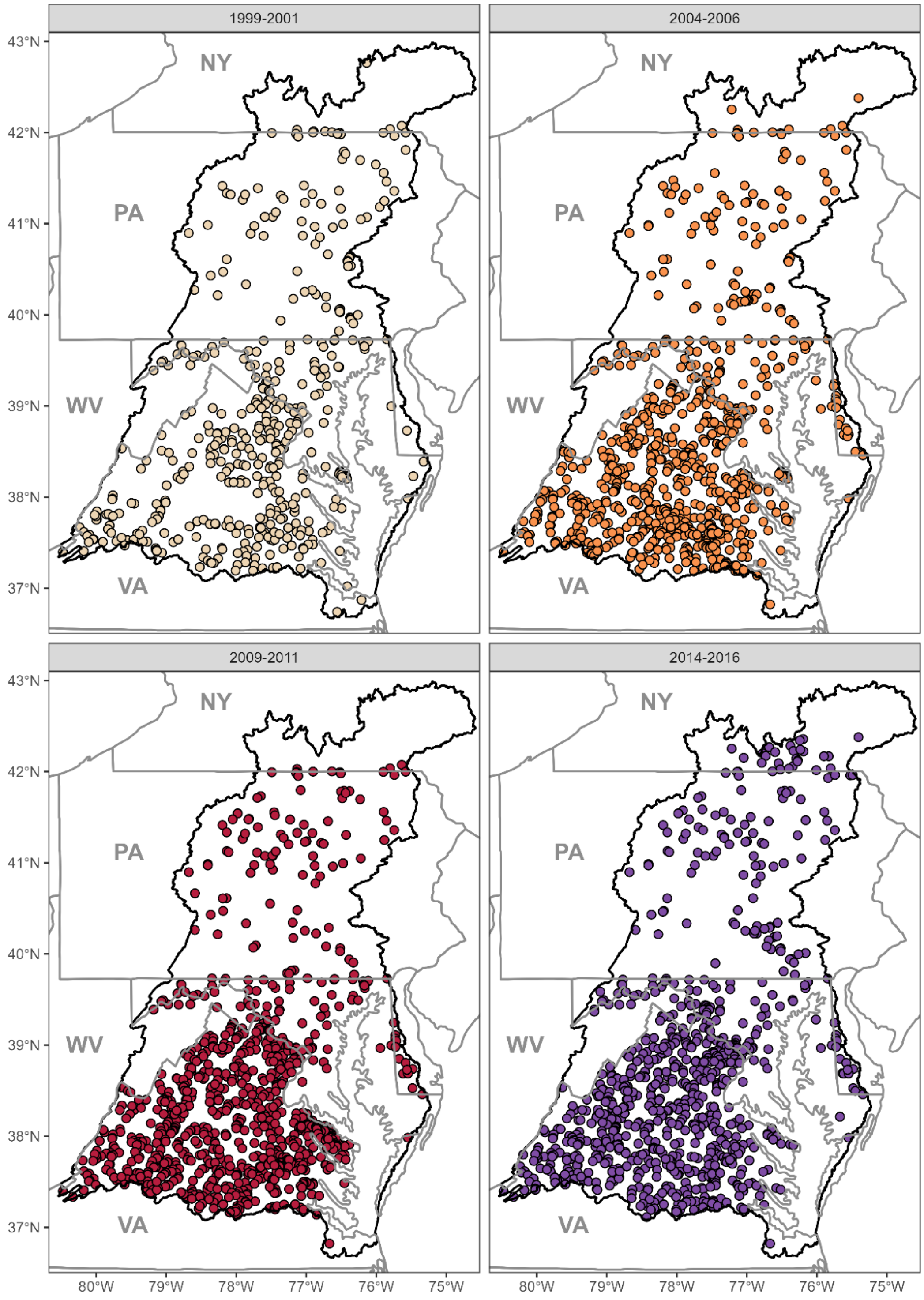

Figure SI-1. Spatial distribution of the testing and training sites used in the random forest regression model across the four time periods. State abbreviations are included for select state names.

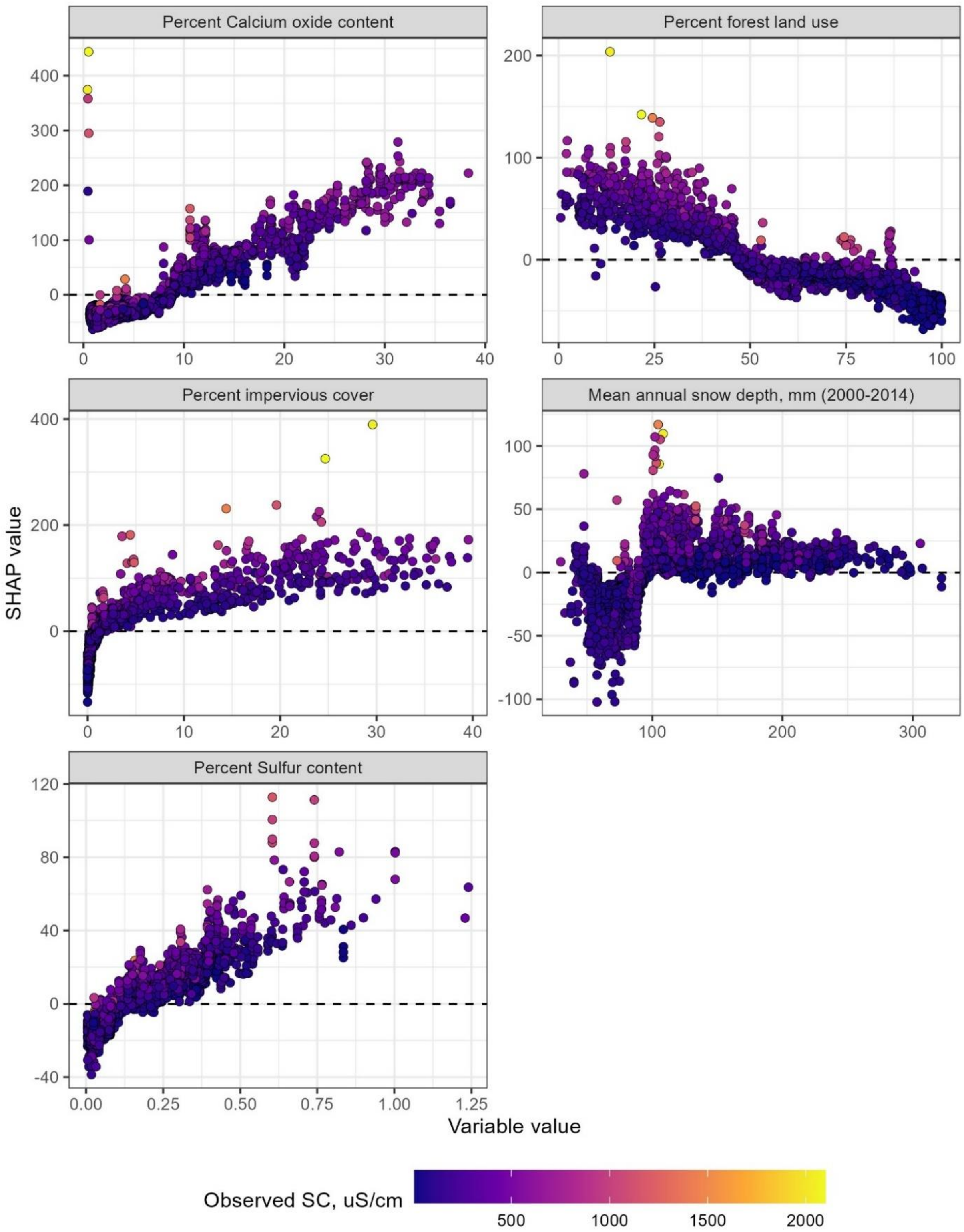

Figure SI-2. Feature contribution (SHAP value) plots for the five most important variables in the random forests model. Points are colored by the observed median annual SC value. See section 2.4 of the methods for more details.

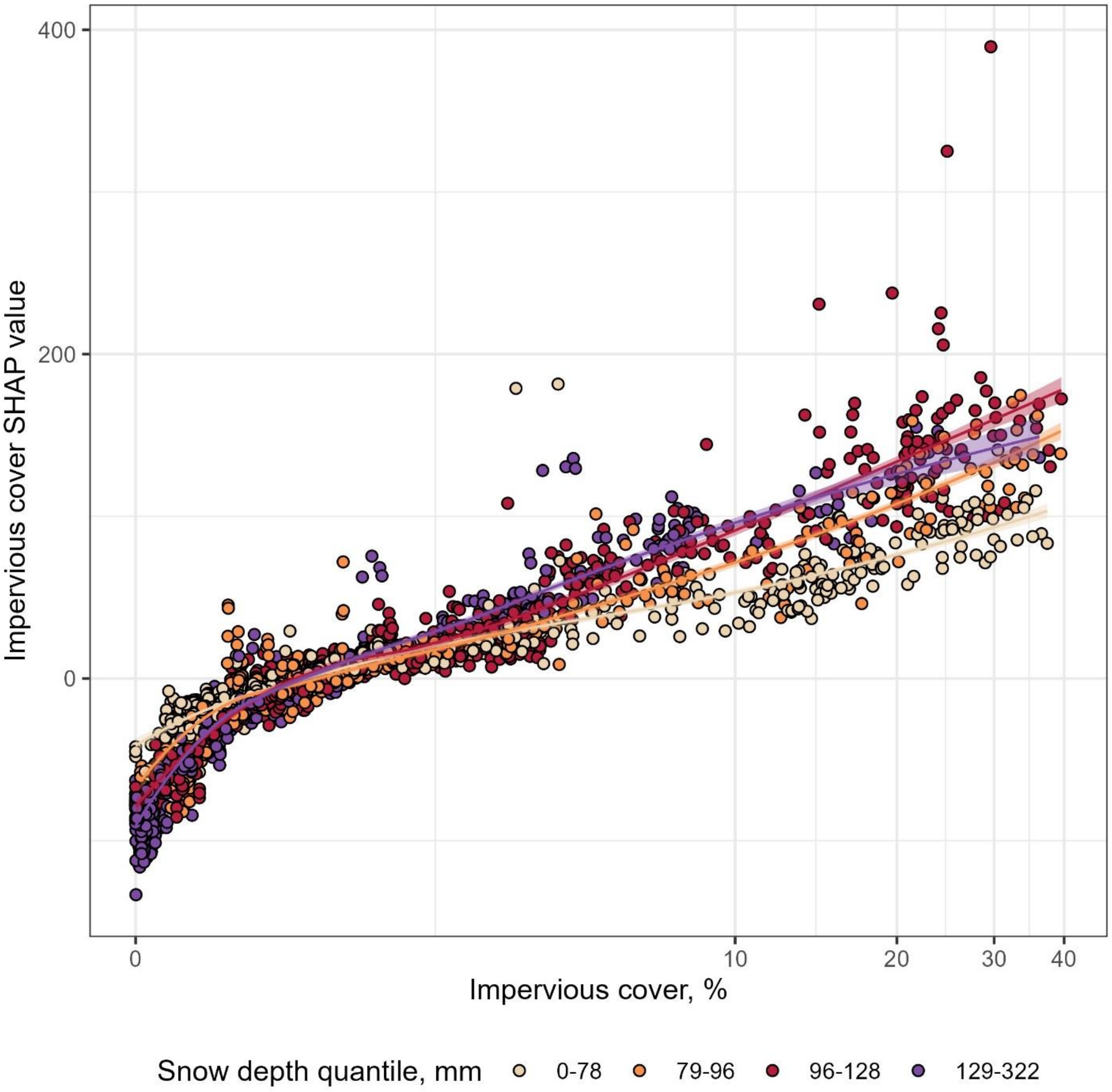

Figure SI-3. Version of Figure 2c with axes fully extended (Impervious cover SHAP values as a function of impervious cover and snow depth). Lines denote a loess smoothing function and shaded areas around the lines represent 95% confidence intervals.

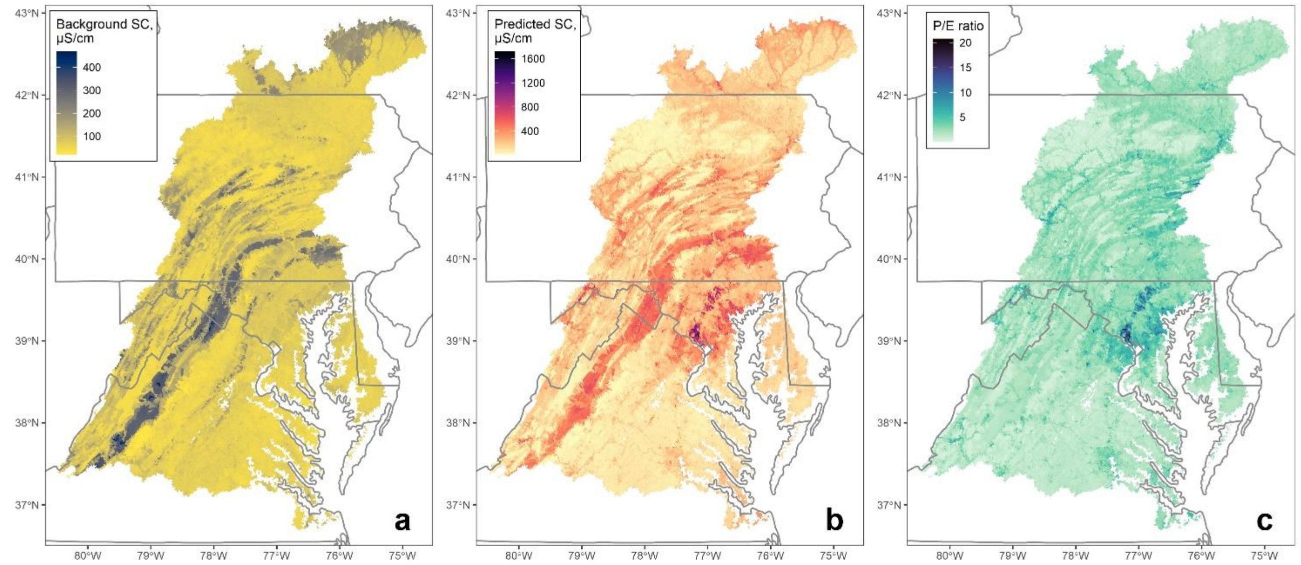

Figure SI-4. Spatial distribution of (**a**) long-term average background (or expected) specific conductance (SC) from Olson and Cormier (2019); (**b**) predicted median annual SC from this study; and (**c**) Predicted SC/expected SC (P/E) ratios (computed by dividing predicted median annual SC values by estimated background SC) for all modeled stream reaches in the Chesapeake Bay watershed for the 2014-2016 time period. A P/E ratio greater than 1 indicates the predicted SC is higher than background SC.

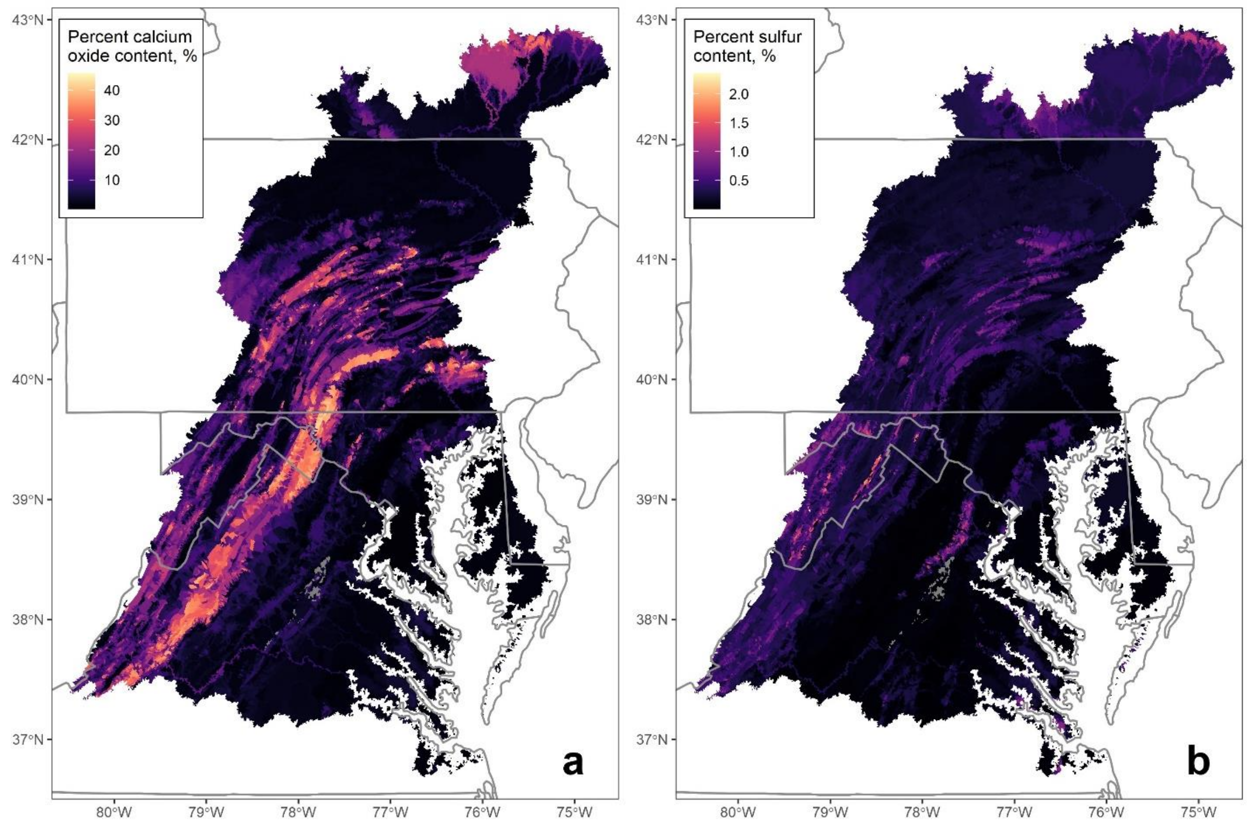

Figure SI-5. Spatial distribution of (**a**) lithologic calcium oxide content and (**b**) lithologic sulfur content in the Chesapeake Bay watershed.

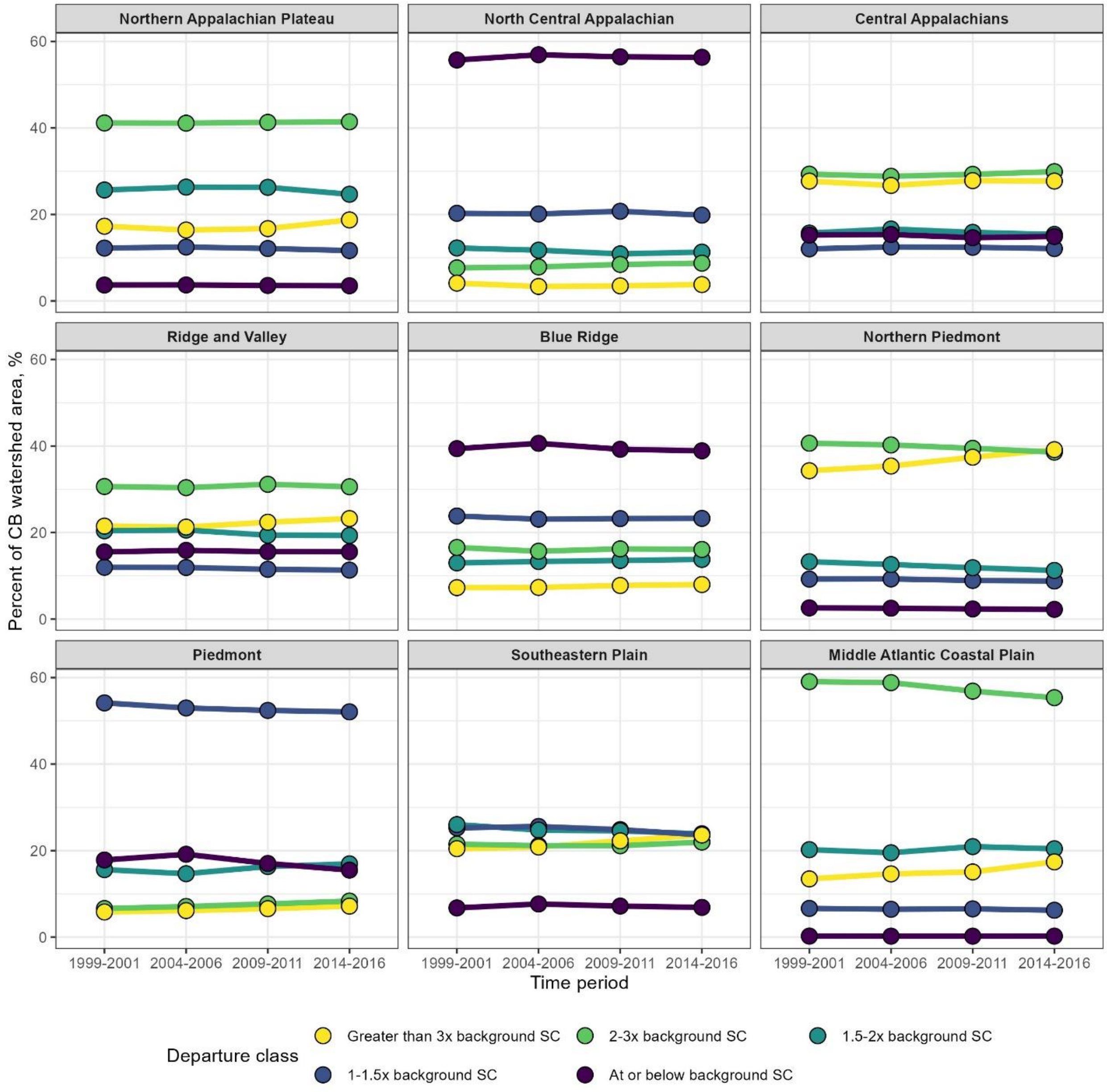

Figure SI-6. Percent of the area within each of the nine major ecoregions that fell into each of the five departure classes across the four time periods. Ecoregions are ordered from north to south.

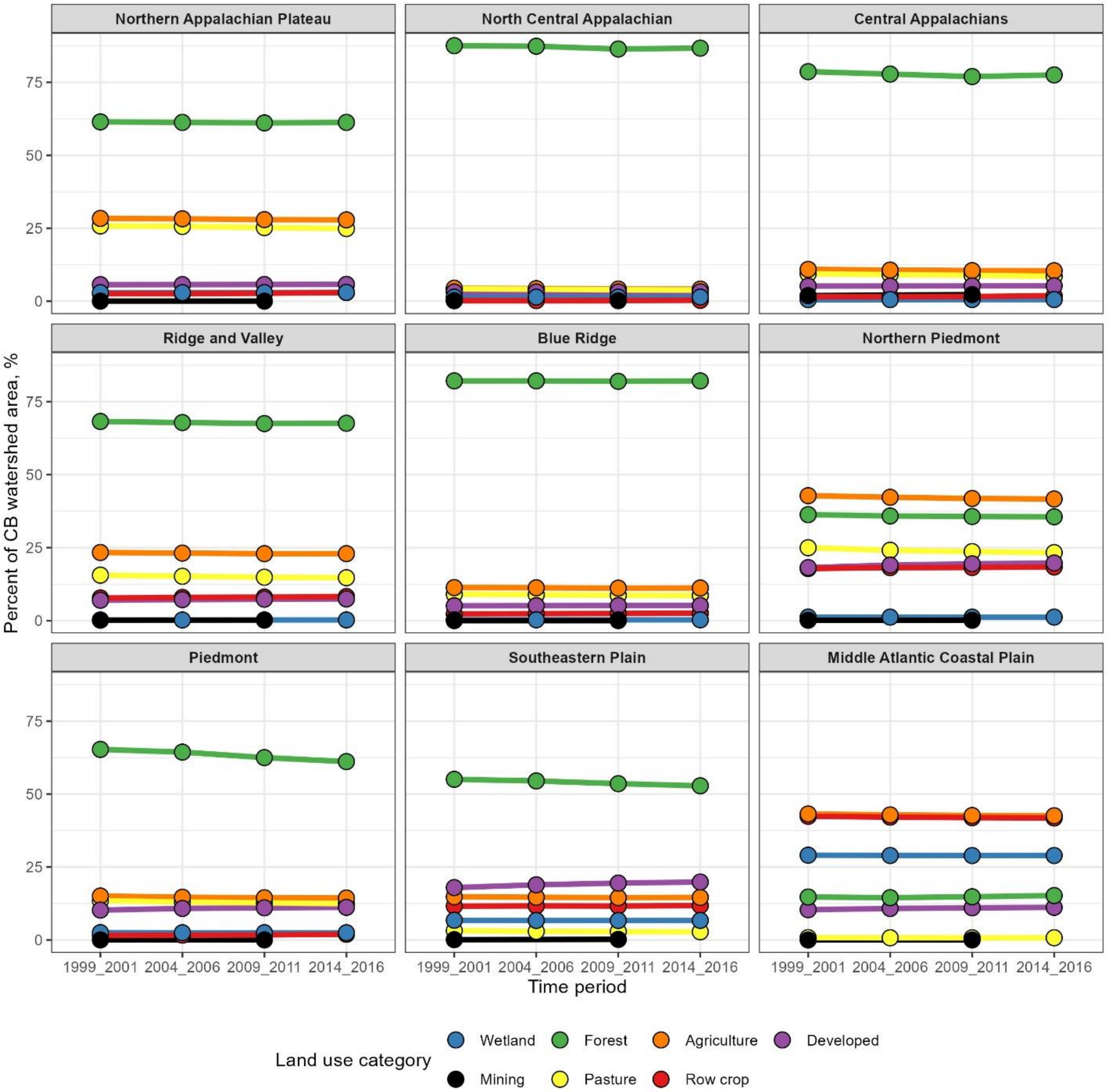

Figure SI-7. Land use and land use change for the four time periods in each of the nine major ecoregions. Note: Percent agriculture land use is the sum of percent row crop land use and percent pasture land use.

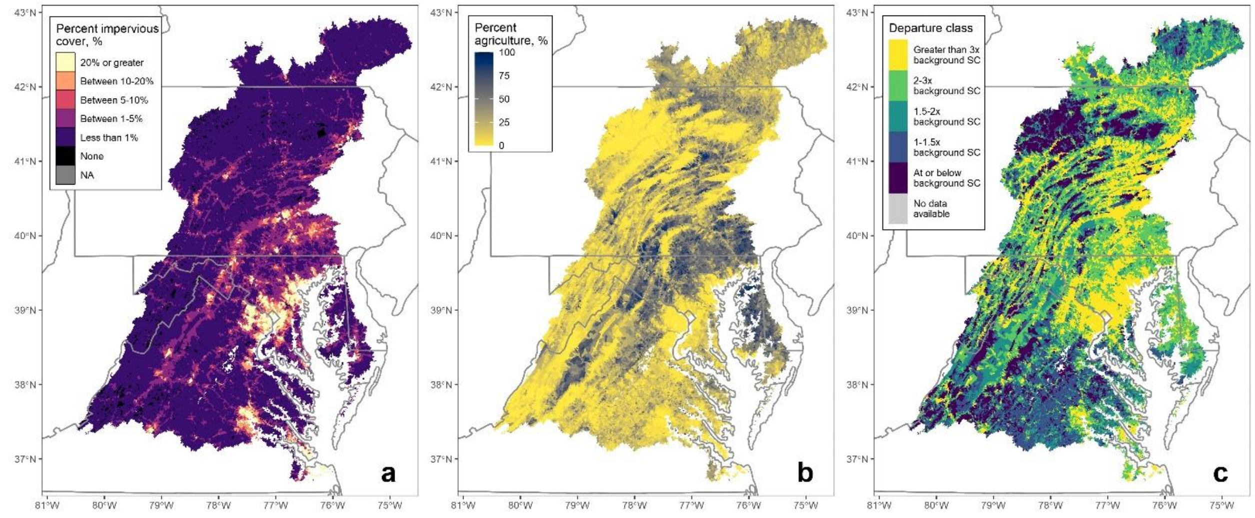

Figure SI-8. Spatial distribution of (**a**) percent impervious cover; (**b**) percent agriculture; and (**c**) departure classes for the 2014-2016 time period in the Chesapeake Bay watershed.

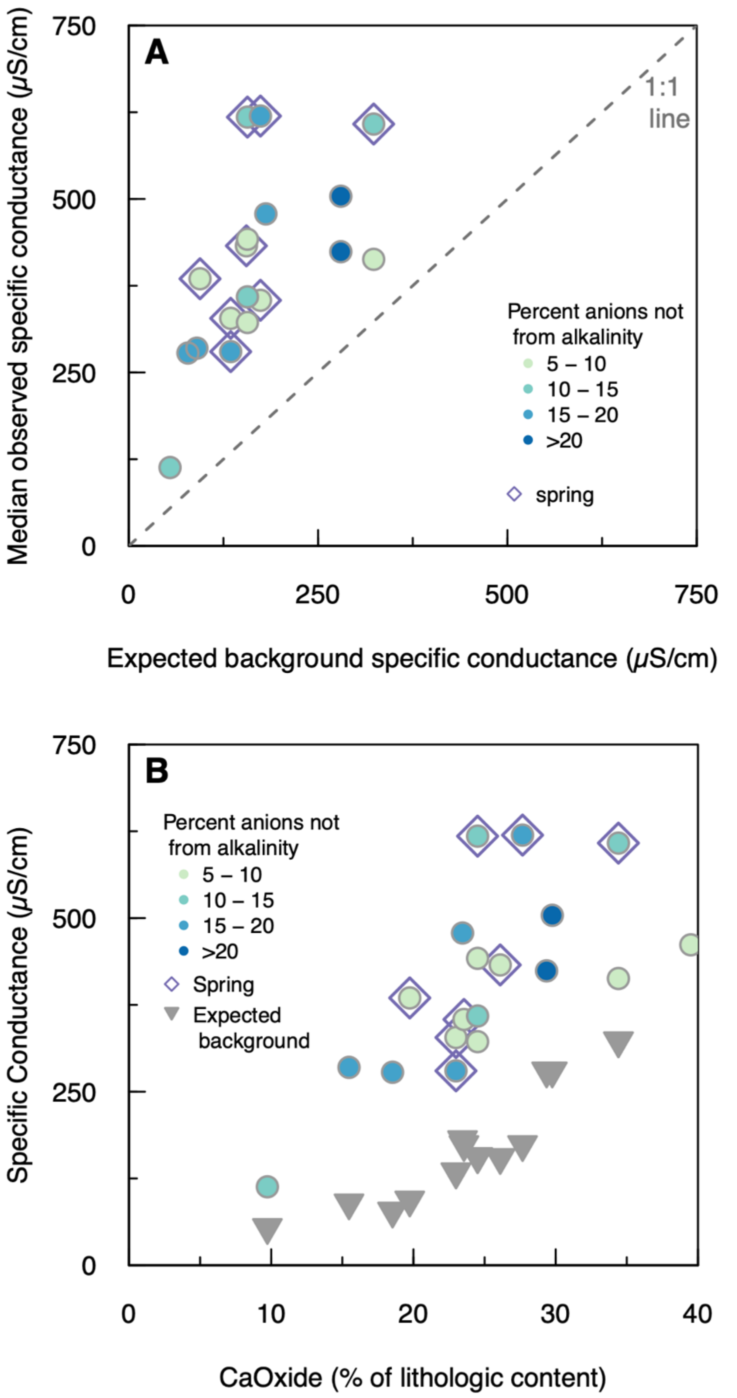

Figure SI-9: (**A**) Observed median specific conductance (SC) versus expected background SC from Olson and Cormier (2019, see also Table SI-6). Colors represent the percentage of the observed anion charge from chloride, nitrate, and sulfate (sources other than alkalinity) with no sites <5%. A threshold of 10% of anion charge from sources other than alkalinity has been proposed for separating waters that have been minimally affected by anthropogenic activity from those with substantial effects (Krawczyk & Ford, 2006). (**B**) SC versus CaOxide for streams and springs from the Chesapeake Bay Watershed, as well as the average of several forested sites

in France plotted at ~40% CaOxide (Table SI-6). The two points at ~29.5% CaOxide are for the same stream from 1993–97 and 2018–22 with slightly lower and higher values than actual for CaOxide plotted for ease of viewing (see Table SI-6). CaOxide = Percent mean lithological calcium oxide content. See Table SI-1 for more details on variable definitions.

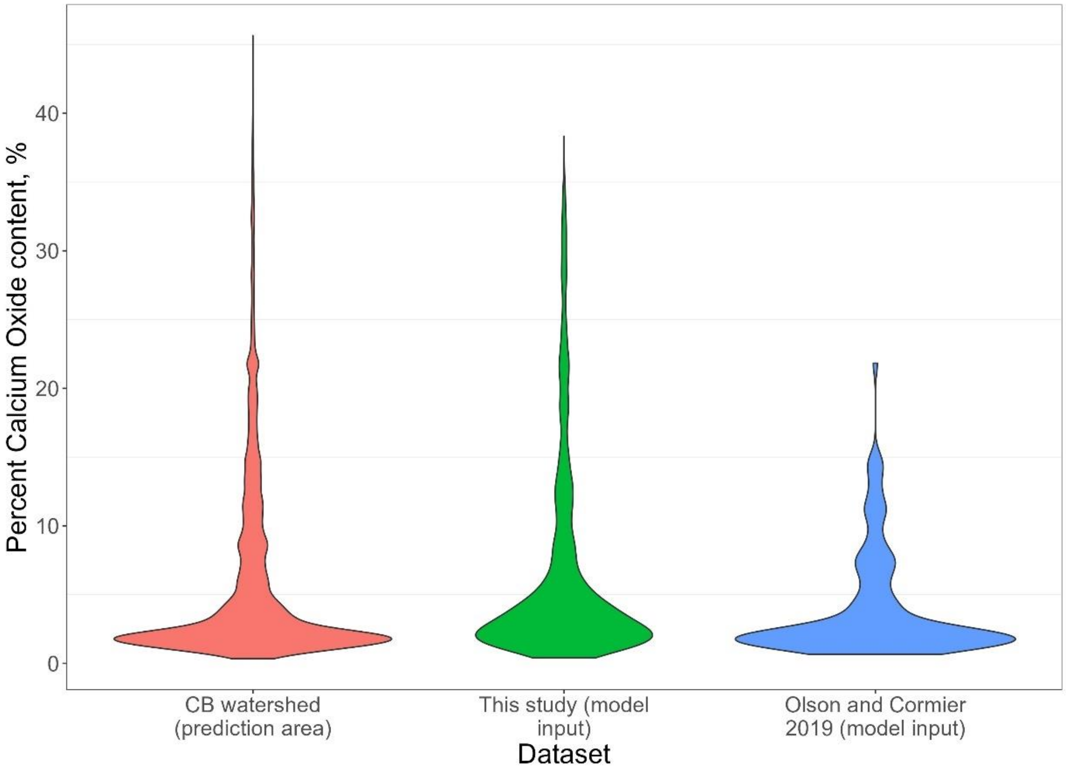

Figure SI-10. Distribution of lithological calcium oxide (CaOxide) content in the Chesapeake Bay watershed, the observations used in the analysis for this study, and the observations used in Olson and Cormier (2019).

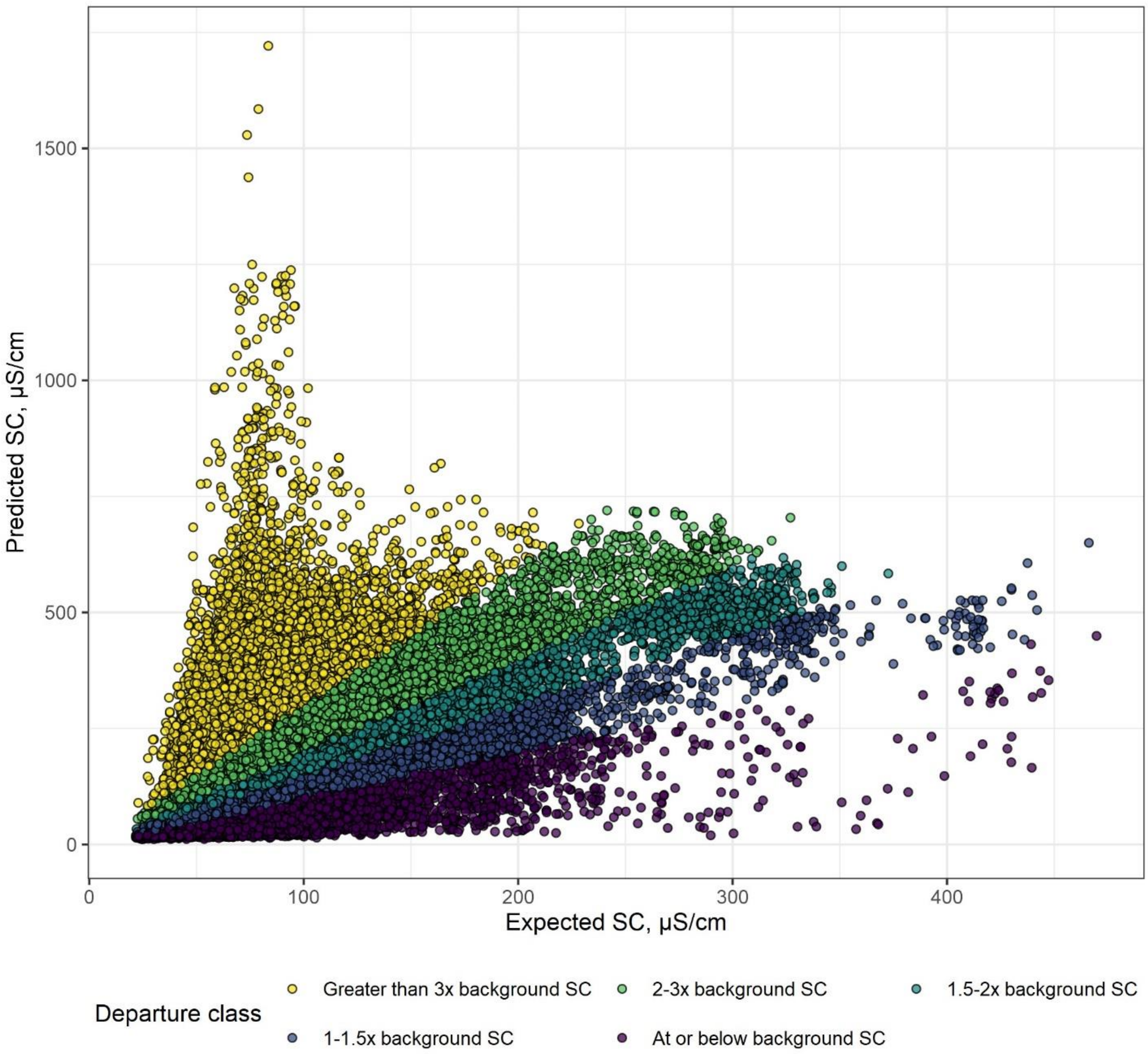

Figure SI-11. Expected (background) specific conductance (SC) vs. predicted SC for all modeled reaches in the CBW for the 2014-2016 time period. Colors indicate the departure class, which is based on the predicted SC/expected SC (P/E) ratio.

Table SI-1: Predictor variables considered or used in the random forest model. Table includes the short and full name of the variable, units, its status as a static or time-varying variable, the source of the summarized data for the NHD v2.1 stream network and the original data source. A justification for dropping the variable is provided for variables that were not selected for the final model. (See separate file titled “Table_SI_1.xlsx”)

Table SI-2. Summary statistics for SC records (i.e., median annual SC observations, µS cm^{-1}) across the four time periods used in the random forests model.

Time Period	Number of records	Mean	Median	Minimum	Maximum
1999-2001	453	185	126	8	1042
2004-2006	853	178	127	9	971
2009-2011	1017	179	116	9	1210
2014-2016	915	213	155	9	2099

Table SI-3: Total area (in km^2) and percent area for the SC departure classes in the study area (non-tidal portion of the Chesapeake Bay watershed) across the four time periods.

Departure class	Metric	1999-2001	2004-2006	2009-2011	2014-2016
Greater than 3x background SC	Area, km^2	29,678	29,608	31,065	32,838
Greater than 3x background SC	Area, %	19.0%	18.9%	19.9%	21.0%
2-3x background SC	Area, km^2	46,548	46,258	46,611	46,277
2-3x background SC	Area, %	29.8%	29.6%	29.8%	29.6%
1.5-2x background SC	Area, km^2	29,910	29,707	29,145	28,615
1.5-2x background SC	Area, %	19.1%	19.0%	18.6%	18.3%
1-1.5x background SC	Area, km^2	27,351	27,207	26,742	26,166
1-1.5x background SC	Area, %	17.5%	17.4%	17.1%	16.7%
At or below background SC	Area, km^2	22,661	23,368	22,585	22,251
At or below background SC	Area, %	14.5%	14.9%	14.4%	14.2%

Table SI-4: Total area (in km^2) and percent area for different land use classes in the study area (non-tidal portion of the Chesapeake Bay watershed) across the four time periods. NA = no data available.

Land use category	Metric	1999-2001	2004-2006	2009-2011	2014-2016
Forest	Area, km^2	95,794	95,160	94,365	94,261
	Area, percent	61.2%	60.8%	60.3%	60.3%
Agriculture	Area, km^2	37,482	37,084	36,730	36,666
	Area, percent	24.0%	23.7%	23.5%	23.4%
Pasture	Area, km^2	23,865	23,294	22,822	22,497
	Area, percent	15.3%	14.9%	14.6%	14.4%
Row crop	Area, km^2	13,617	13,790	13,907	14,169
	Area, percent	8.7%	8.8%	8.9%	9.1%
Developed	Area, km^2	14,296	14,815	15,120	15,311
	Area, percent	9.1%	9.5%	9.7%	9.8%
Wetland	Area, km^2	4,867	4,852	4,854	4,879
	Area, percent	3.1%	3.1%	3.1%	3.1%
Mining	Area, km^2	279	NA	322	NA
	Area, percent	0.18%	NA	0.21%	NA

Table SI-5. Ecoregion land use characteristics for the most recent time period (2014-2016). Year listed on top indicates the actual year for the land use category. Please note that percent mining data for the most recent time period was represented using data from 2012 (the most recent available percent mining data).

		2014-2016					2012
**Ecoregion**	**Metric**	**Pasture**	**Row crop**	**Developed**	**Forest**	**Wetland**	**Mining**
Northern Appalachian Plateau	Area, km^2	6,154	735	1,427	15,145	749	10.1
	Area, %	24.9%	3.0%	5.8%	61.3%	3.0%	0.04%
North Central Appalachian	Area, km^2	434	36	363	9,819	162	21.6
	Area, %	3.8%	0.3%	3.2%	86.7%	1.4%	0.2%
Central Appalachians	Area, km^2	512	115	317	4,648	34	136.5
	Area, %	8.5%	1.9%	5.3%	77.5%	0.6%	2.3%
Ridge and Valley	Area, km^2	7,515	4,218	3,791	34,581	121	91.8
	Area, %	14.7%	8.2%	7.4%	67.6%	0.2%	0.2%
Blue Ridge	Area, km^2	561	166	337	5,342	18	0.4
	Area, %	8.6%	2.6%	5.2%	82.1%	0.3%	0.01%
Northern Piedmont	Area, km^2	4,761	3,754	4,034	7,268	244	25.9
	Area, %	23.3%	18.4%	19.7%	35.6%	1.2%	0.1%
Piedmont	Area, km^2	1,858	292	1,670	9,133	373	6.4
	Area, %	12.4%	2.0%	11.2%	61.1%	2.5%	0.04%
Southeastern Plain	Area, km^2	336	1,409	2,375	6,316	801	24.9
	Area, %	2.8%	11.8%	19.9%	52.8%	6.7%	0.2%
Middle Atlantic Coastal Plain	Area, km^2	60	3,303	883	1,203	2,287	2.0
	Area, %	0.8%	41.8%	11.2%	15.2%	28.9%	0.03%

Table SI-6: Comparison of predicted background specific conductance (SC, µS cm^{-1}) versus observed data. NA = no data available. Alk = alkalinity

Site name	USGS site_no	ComID	Predicted background SC[a]	25% actual SC	Median actual SC	75% actual SC	Non-alk anion (%)	Median alkalin. (µeq/L)	Median Cl + NO3 + SO4 (µeq/L)	CaOxide (wt %)	Citation for obs. data
Springs 9-11	NA	8140418	134.6	NA	328	NA	5.9	2901	259.6	22.99	1[b]
Jura Mtns	NA	NA	NA	433	462	500	5.9	4758	329.5	39.50	2[c]
Springs 20-22	NA	8140320	94.1	NA	385	NA	6.8	3622	436.5	19.74	1[b]
Springs 13-14	NA	8140310	155.3	NA	433	NA	7.6	3843	540.6	26.1	1[b]
War Branch at Oakwood, Va	01632866	8441089	156.7	278	322	335	7.6	3237	303.8	24.51	3,4
Big Spring Near Lacey Spring, Va	01632835	8441323	173.9	322	354	397	8.5	3487	384.8	23.55	3,4
Smith Creek at Route 811 Near Lacey Spring, Va	01632837	8442071	323.4	347	413	428	9.3	4057	497.0	34.41	3,4
War Branch at Mouth at Tenth Legion, Va	01632882	8441089	156.7	415	442	463	9.8	4196	554.1	24.51	3,4
Mountain Run at Rt 620 Near Lacey Spring, Va	01632833	8442049	54.5	95	113	156	10.5	993	168.8	9.73	3,4
War Branch Near Athlone, VA	01632874	8441089	156.7	332	359	372	10.8	3377	517.9	24.51	3,4
Smith Creek at Spring Near Harrisonburg, Va	01632802	8442071	323.4	595	608	619	12.6	5775	968.6	34.41	3,4
War Branch Tributary Near Tenth Legion	01632878	8441089	156.7	601	618	651	13.9	5515	1021	24.51	3,4
Lacey Spring at Lacey Spring, VA	0163285551	8441319	173.9	612	620	651	16.3	5595	1318	27.67	3,4
Fishing Crk near Cedar Springs	01548075	8139388	89.9	244	285	339	20.8	2138	772.6	15.47	4
Smith Crk at New Market	01632900	8441303	181	397	479	519	18.5	4136	1105	23.45	3,4
Elk Creek at Spring Bank near Millheim, PA	01554665	4519174	78.1	227	278	311	17.4	2348	599.8	18.53	4
Springs 7-8	NA	8140418	134.6	NR	280	NA	21	2467	661.2	22.99	1[b]
Muddy Creek at Mount Clinton, Va 1993-97	01621050	5908085	280	392	424	449	14.8	3697	747.8	29.56	4[d]
Muddy Creek 2018-22	01621050	5908085	280	434	504	539	18.1	4496	1130.8	29.56	4[d]
Muddy Creek all	01621050	5908085	280	418.25	469	525	16.9	3997	914.6	29.56	4[d]

[a] Cormier et al. (2021)
[b] Chloride, nitrate, and sulfate concentrations for samples without reported values were calculated as 50% of the minimum value reported for springs.
[c] Only sites ≤650 m in elevation were included (see SI text for details) and SC values were calculated using PHREEQ and code (McCleskey, 2018).
[d] The Muddy Creek values for 1993–97 and 2018–22 are shown on Figure SI-2 but the overall average is not.

Citations for observed data in table SI-6
(1) Langmuir D., 1971; (2) Calmels et al., 2014; (3) Hyer et al., 2016; (4) USGS, 2023

References

Bock, A.R., Falcone, J.A., Oelsner, G., and Baker, N.T., 2018, Estimates of Road Salt Application across the Conterminous United States, 1992-2019 (ver. 2.0, August 2023): U.S. Geological Survey data release, https://doi.org/10.5066/P96IX385

Bolton, D. W. (1998), Ground-water quality in the Piedmont region of Baltimore County, Maryland. Report of Investigations 66, Professional Paper, 58 pp, Maryland Geological Survey, Baltimore, MD. No DOI

Calmels D., Gaillardet J., and François L. (2014), Sensitivity of carbonate weathering to soil CO2 production by biological activity along a temperate climate transect. *Chemical Geology* 390, 74-86. DOI: 10.1016/j.chemgeo.2014.10.010

Chesapeake Bay Program. 2004. Chesapeake Bay Program Analytical Segmentation Scheme Revisions, Decisions and Rationales 1983–2003. Prepared by the Chesapeake Bay Program Monitoring and Analysis Subcommittee Tidal Monitoring and Analysis Workgroup Annapolis, Maryland. October 2004. Available from: https ://www.chesapeakebay.net/content/publications/cbp_13272.pdf. Accessed: 1 May 2024.

Cormier S., Wharton C., Olson J., U.S. EPA Freshwater Explorer. Version 0.1. United States Environmental Protection Agency. Accessed July 2021. https://arcg.is/KHb9S.

Dewitz, J., and U.S. Geological Survey, 2021, National Land Cover Database (NLCD) 2019 Products (ver. 2.0, June 2021): U.S. Geological Survey data release, https://doi.org/10.5066/P9KZCM54

Falcone, J.A., 2015, U.S. conterminous wall-to-wall anthropogenic land use trends (NWALT), 1974–2012: U.S. Geological Survey Data Series 948, 33 p. plus appendixes 3–6 as separate files, http://dx.doi.org/10.3133/ds948

Fanelli, R.M., Sekellick, A.J., and Hamilton, W.B., 2023, Compilation of multi-agency specific conductance observations for streams within the Chesapeake Bay watershed, U.S. Geological Survey data release, https://doi.org/10.5066/P98O2HQJ.

Hill, R. A., Weber, M. H., Leibowitz, S. G., Olsen, A. R. & Thornbrugh, D. J. 2016. The Stream-Catchment (StreamCat) Dataset: A Database of Watershed Metrics for the Conterminous United States. JAWRA Journal of the American Water Resources Association 52, 120–128.

Hyer K. E., Denver J. M., Langland M. J., Webber J. S., Bohlke J. K., Hively W. D., and Clune J. W. (2016) Spatial and temporal variation of stream chemistry associated with contrasting geology and land-use patterns in the Chesapeake Bay watershed: Summary of results from Smith Creek, Virginia; Upper Chester River, Maryland; Conewago Creek, Pennsylvania; and Difficult Run, Virginia, 2010–2013: US Geological Survey Scientific Investigations Report 2016-5093. U.S. Geological Survey.

Krawczyk W. E. and Ford D. C. (2006) Correlating specific conductivity with total hardness in limestone and dolomite karst waters. *Earth Surface Processes and Landforms* **31**, 221-234. DOI: 10.1002/esp.1232

Langmuir D. (1971) The geochemistry of some carbonate ground waters in central Pennsylvania. *Geochimica et Cosmochimica Acta* 35, 1023-1045. DOI: 10.1016/0016-7037(71)90019-6

Lerman, A., and L. Wu (2008), Kinetics of global geochemical systems, in *Kinetics of Water-Rock Interaction*, edited by S. L. Brantley, J. D. Kubicki and A. F. White, pp. 655-736, Springer-Kluwer.

McCleskey, R. B. (2018), Calculated specific conductance using PHREEQCI: U.S. Geological Survey software release, https://doi.org/10.5066/F7M907VD.

Nalenz, M., Rodemann, J., and Augustin, T. Learning De-biased Regression Trees and Forests from Complex Samples. *Mach. Learn.* **2024**. DOI: 10.1007/s10994-023-06439-1

Nelms, D. L., and J. Moberg, R. M. (2010), Hydrogeology and groundwater availability in Clarke County, Virginia: U.S. Geological Survey Scientific Investigations Report 2010–5112. DOI: 10.3133/sir20105112

Olson, J. R., and S. M. Cormier (2019), Modeling spatial and temporal variation in natural background specific conductivity, *Environ Sci Technol*, 53, 4316-4325.

Parkhurst, D. L., and C. A. J. Appelo (2013), Description of input and examples for PHREEQC version 3--A computer program for speciation, batch-reaction, one-dimensional transport, and inverse geochemical calculations: Vol. book 6. U.S. Geological Survey. https://pubs.usgs.gov/tm/06/a43, Water-Resources Investigations Report Rep. WRIR 99-4259, U.S. Geological Survey, Washington, DC.

Sekellick, A.J., 2017, Nitrogen and phosphorus from fertilizer and manure in the Chesapeake Bay watershed, 1950-2012: U.S. Geological Survey data release, https://doi.org/10.5066/F7TQ6011.

Toran, L., J. H. Tancredi, E. K. Herman, and W. B. White (2006), Conductivity and sediment variation during storms as evidence of pathways to karst springs, in *Perspectives on Karst Geomorphology, Hydrology, and Geochemistry—A Tribute Volume to Derek C. Ford and William B. White*, edited, 404, pp. 169-176, Geological Society of America. DOI: 10.1130/2006.2404(14)

Toran, L., K. Gross, and Y. Yang (2009), Effects of restricted recharge in an urban karst system, *Environmental Geology*, 58, 131-139. DOI: 10.1007/s00254-008-1500-0

Toth, D., and Eltinge, J. L. 2011. Building Consistent Regression Trees From Complex Sample Data. *J. Am. Stat. Assoc.* 2011, *106* (496)*, 1626-1636.* http://www.jstor.org/stable/23239564

U.S. Geological Survey, 2023, USGS water data for the Nation: U.S. Geological Survey National Water Information System database, accessed April 4, 2024, at https://doi.org/10.5066/F7P55KJN

Vesper, D. J., and E. K. Herman (2020), Common spring types in the Valley and Ridge Province: There is more than karst, *Environ Eng Geosci*, 26(3), 345-358. DOI: 10.2113/EEG-2321

Wieczorek, M. E., Jackson, S. E. & Schwartz, G. E. 2018. Select Attributes for NHDPlus Version 2.1 Reach Catchments and Modified Network Routed Upstream Watersheds for the Conterminous United States. U.S. Geological Survey data release https://doi.org/10.5066/F7765D7V
